# Peptide Assembly on the Membrane Determines the HIV-1 Inhibitory Activity of Dual-Targeting Fusion Inhibitor Peptides

**DOI:** 10.1038/s41598-019-40125-4

**Published:** 2019-03-01

**Authors:** Maria J. Gomara, Yolanda Perez, Javier P. Martinez, Ramon Barnadas-Rodriguez, Anke Schultz, Hagen von Briesen, Alex Peralvarez-Marin, Andreas Meyerhans, Isabel Haro

**Affiliations:** 1 Unit of Synthesis & Biomedical Applications of Peptides, Institute of Advanced Chemistry of Catalonia (IQAC-CSIC), Jordi Girona 18-26, 08034 Barcelona, Spain; 2 Nuclear Magnetic Resonance Facility, IQAC-CSIC, Barcelona, Spain; 3 Infection Biology Laboratory, Department of Experimental and Health Sciences, Universitat Pompeu Fabra, Barcelona, Spain; 4 ICREA, Pg. Lluís Companys 23, 08010 Barcelona, Spain; 5 Unit of Biophysics, Department of Biochemistry & Molecular Biology, Universitat Autonoma of Barcelona, 08193 Cerdanyola del Vallés, Spain; 6 Fraunhofer Institute for Biomedical Engineering IBMT, Joseph-von-Fraunhofer-Weg 1, Sulzbach, Germany

## Abstract

Novel strategies in the design of HIV-1 fusion/entry inhibitors are based on the construction of dual-targeting fusion proteins and peptides with synergistic antiviral effects. In this work we describe the design of dual-targeting peptides composed of peptide domains of E2 and E1 envelope proteins from Human Pegivirus with the aim of targeting both the loop region and the fusion peptide domains of HIV-1 gp41. In a previous work, we described the inhibitory role of a highly conserved fragment of the E1 protein (domain 139–156) which interacts with the HIV-1 fusion peptide at the membrane level. Here, two different dual-targeting peptides, where this E1 peptide is located on the N- or the C-terminus respectively, have been chemically synthesized and their antiviral activities have been evaluated with HIV pseudotyped viruses from different clades. The study of the functional behaviour of peptides in a membranous environment attending to the peptide recognition of the target sites on gp41, the peptide conformation as well as the peptide affinity to the membrane, demonstrate that antiviral activity of the dual-targeting peptides is directly related to the peptide affinity and its subsequent assembly into the model membrane. The overall results point out to the necessity that fusion inhibitor peptides that specifically interfere with the N-terminal region of gp41 are embedded within the membrane in order to properly interact with their viral target.

## Introduction

Similarly as innovations in HIV-1 immunotherapy are focused on the design and engineering of bispecific antibodies with two antigen-binding variable fragments of immunoglobulins that recognize two separate antigens, (reviewed by Ferrari *et al*.^1^), novel strategies in the design of HIV-1 fusion/entry inhibitors are based on the construction of dual-targeting fusion proteins and peptides with synergistic antiviral effects (reviewed by Castro *et al*.^2^). Examples of recombinant protein chimeras have been recently reported. Thus, recombinant protein chimeras produced by fusing lectins to peptides derived from the membrane-proximal external region of gp41 have been designed to target simultaneously gp120 and gp41 domains causing lytic inactivation of HIV-1^3,4^. Moreover, in order to design HIV-1 fusion inhibitors with targeting properties to the precise site of HIV-1 entry, the C34 peptide has been conjugated to anchoring molecules over-expressed on the cell surface such as co-receptors CCR5 or CXCR4. These recombinant protein chimeras have been expressed on the surface of T cell lines and primary CD4+ T cells, conferring particularly potent, broad and durable protection from HIV-1 infection to primary CD4+ T cells *in vitro* and in humanized mice^5^. In addition to recombinant proteins, dual-targeting peptide HIV-1 fusion inhibitors have also been described with the ability of binding simultaneously and cooperatively to different gp41 domains^6–8^. The rationale for the design of chimeric peptide fusion inhibitors is based on the sequential nature of the HIV-1 virus-cell fusion process characterized by the appearance of multiple targets that are susceptible to inhibition. Thus, the combination of two different peptide fusion inhibitors in the same molecule theoretically implies a synergistic fusion inhibitory activity as well as a greater probability of avoiding the appearance of resistant virus.

GB virus type C (GBV-C), recently renamed as Human Pegivirus (HPgV)^9^, can be considered as a symbiont or commensal of humans since infections provide a beneficial effect on survival in HIV-1 positive subjects^10^. Previous work of our group demonstrated the antiviral properties of chimeric molecules composed of an E2 region from GBV-C that targets the gp41 loop region and the peptide sequence of VIR576 that interacts with the gp41 fusion peptide (FP), both described as HIV-1 inhibitors^7^. Following the same approach, in this work the E2 sequence has been combined with an 18-mer domain from the E1 protein that interacts with the gp41 fusion peptide at the membrane level and has a broad spectrum activity against HIV-1, as we previously reported^11^. Trying to improve this anti-HIV-1 activity, two dual targeting peptides (DT-peptides), where the E1 peptide is on the N- or the C-terminus respectively, have been synthesized. The antiviral activities of the DT-peptides have been evaluated with HIV pseudotyped viruses from different clades.

Since HIV-1 gp41 glycoprotein is confined between the cellular and the viral membranes, the study of the physicochemical processes involved at this interface is essential to understand the mode of action of fusion inhibitor peptides^12–14^. As it has been previously reported, the interaction of fusion inhibitor peptides with biological membranes may be related to their inhibition efficiency^15–17^. Based on this background, in this work conformational and biophysical assays using model membranes have been carried out in order to understand better the requirements of DT-peptides for maintaining their functional behaviour as HIV-1 fusion inhibitors.

## Results and Discussion

**Figure 1:**
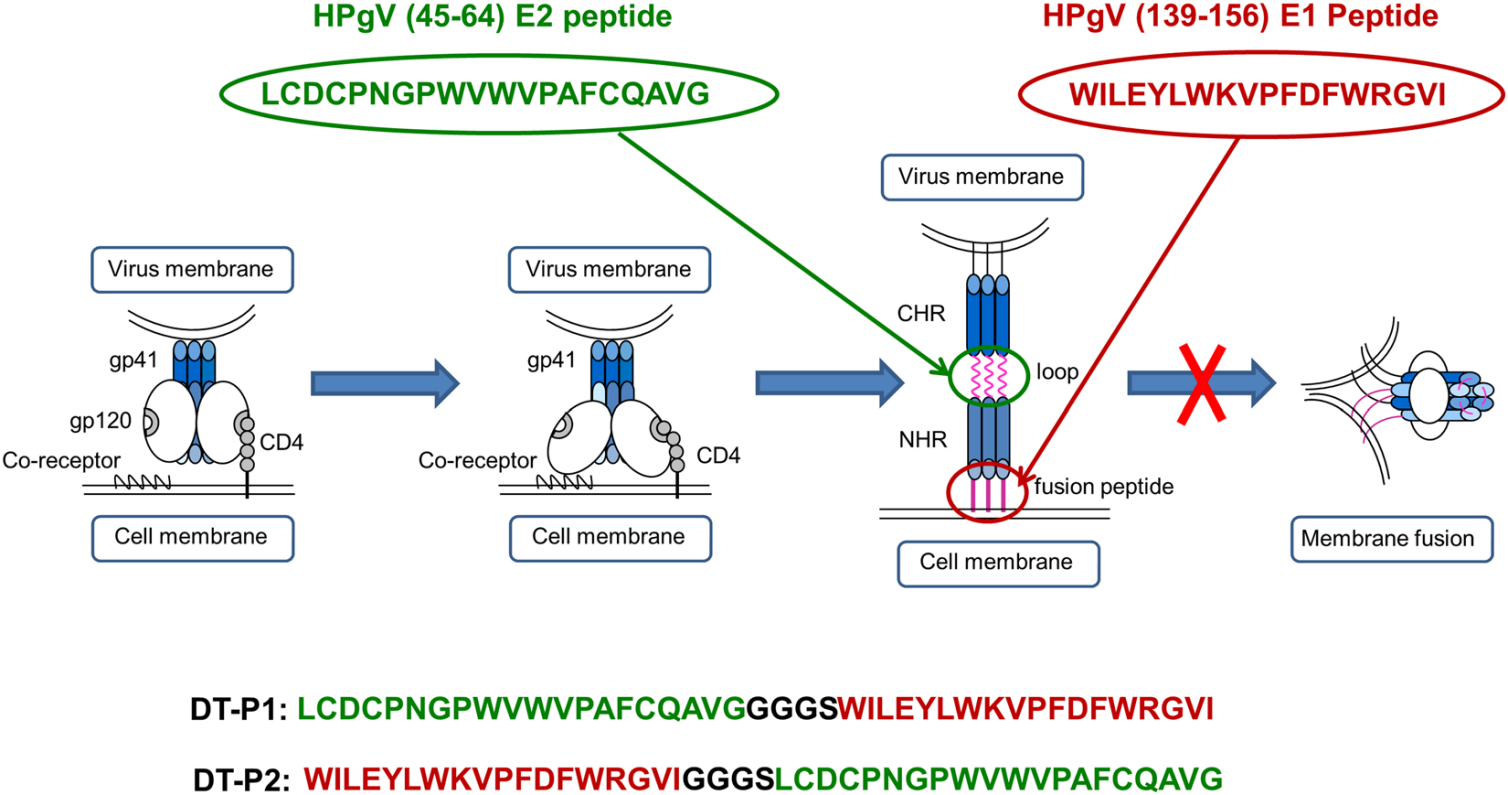
Schematic representation of the corresponding target sites on HIV-1 gp41 glycoprotein of the selected peptide inhibitors: HPgV (45–64) E2 peptide and HPgV (139–156) E1 peptide. Primary sequences of the DT-peptides, DT-P1 and DT-P2, are depicted at the bottom of the figure.

DT-peptides composed of two different sequences from E1 and E2 glycoproteins of the non-pathogenic HPgV have been synthesized with the purpose of targeting two different regions of HIV-1 gp41: the loop and the fusion peptide (FP) (Fig. 1).

To this aim, we have selected the peptide sequence comprising the (45–64) region from the N-terminal part of the E2 protein that has been described as a fusion inhibitor of the late HIV-1 entry steps via interaction with the disulphide loop region of HIV-1 gp41^18^. In addition, we have also selected the (139–156) region of the E1 protein since recent structural studies carried out by our group have demonstrated the interaction of this peptide sequence with the HIV-1 FP at the membrane level interfering with the stabilization of the six-helix bundle formation in a membranous environment^19^. Penetration of both E1 and E2 peptides in vaginal mucosa through their release from polymeric nanoparticles has been recently studied as a preliminary biopharmaceutical evaluation of their potential microbicidal usefulness^20,21^. Based on these two peptide sequences from the HPgV, described as fusion inhibitors, two different DT-peptides have been synthesized combining the sequences in different orientation through a Gly/Ser linker. Dual-targeting peptide 1 (DT-P1) contains the E1 sequence on C-terminus whereas dual-targeting peptide 2 (DT-P2) contains the E1 sequence on N-terminus of the chimeric peptide. The primary structure of DT-peptides is detailed in Fig. 1.

The anti-HIV activities of DT-peptides and E1 were comparatively tested against a set of viral pseudotypes carrying HIV envelopes from HIV subtype A, B, C and A/E (Table 1) in the TZM-bl reporter cell line. Table 2 shows the effective concentration 50 (EC_50_) values of E1, DT-P1 and DT-P2 peptides.

**Table 1:** Plasmids used to produce the HIV pseudotypes that express the described envelope protein. Pseudotypes were produced by plasmid co-transfections in 293T cells as described by Martínez *et al*.^39^.

Type	Name	Tropism	Subtype	NIH cat#	SHORT ID
Backbone	pSG3ΔEnv*			11051	
Env	pHXB2	X4	B	1069	HXB2
Env	pAC10.0	R5	B	11024	AC10
Env	pQ461.e2	R5	A	10460	Q46
Env	pQ23.17	R5	A	10455	Q23
Env	pCAP210.2	R5	C	11317	CAP2
Env	pCM235/GS	R5	A/E	7701	CM235

^*^HIV expression vector that expresses all HIV-1 proteins except Env and Vpu. For all pseudotypes: TZM-bl cells were infected with a virus inoculum corresponding to a RLU/s of 10-fold higher than RLU/s in control cells; RLU/s = Relative Light Units per second.

**Table 2:** EC_50_ values (in µM) of the peptides against a panel of HIV pseudotypes carrying different envelopes.

	E1		DT-P1		DT-P2	
	EC_50_	SEM	EC_50_	SEM	EC_50_	SEM
HXB2	2.49	0.40	7.63	0.88	10.77	1.03
AC10	3.93	0.59	20.18	1.31	14.02	1.15
Q461	2.71	0.43	24.46	1.39	24.49	1.39
Q23	6.65	0.82	21.62	1.34	15.86	1.20
CAP210	1.12	0.30	12.94	0.43	9.40	0.43
CM235	8.78	0.94	18.93	1.28	17.94	1.25

Both DT-peptides demonstrated similar antiviral activities against all pseudotypes in the low micromolar range although they were between 2 and 10 times less active than the single E1 peptide. Neither of the peptides showed cell toxicity at the tested concentrations. To get further on the functional behavior of DT-peptides responsible for the lower activity, conformational and biophysical studies have been carried out. Three different issues on the peptides have been taken into account that might be directly related to the antiviral activity: the peptide recognition of the target sites on gp41, the peptide conformation adopted in a membranous environment and the peptide affinity to the membrane.

### DT-P2 maintains better recognition of both gp41 target sites than DT-P1

**Figure 2:**
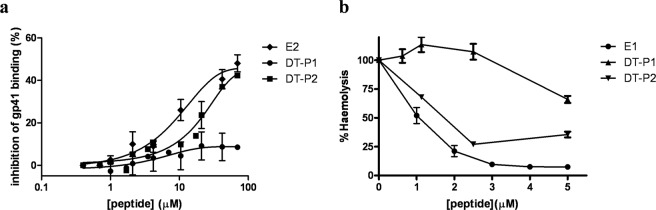
Comparative study of interaction of the DT-peptides with the HIV-1 gp41 target domains: gp41 loop and FP. (**a**) Inhibition of the mAb (F240) binding to immobilized gp41_MN_ induced by DT-peptides and E2 peptide in a competitive assay. (**b**) Inhibitory effect of DT-P1, DT-P2 and E1 peptides on haemolysis induced by the HIV-1 FP. Y axis represents the percentage of haemolysis expressed relative to the total haemolysis caused by the respective HIV-1 FP alone.

The capability of preserving the recognition of target sites on gp41 by both DT-P1 and DT-P2 was first studied. As previously reported^7,18^, to specifically study the target site of the E2 peptide for DT-peptides we have used a competitive immunoassay using the specific gp41-targeting mAb (F240) that recognize the disulfide loop region on gp41 glycoprotein. As it is shown in Fig. 2a, both DT-peptides didn’t inhibit similarly the specific recognition of the truncated recombinant gp41_MN_ by the mAb F240. Although, DT-P2 was able to compete with the mAb F240 in the recognition of the disulfide loop region in a dose-dependent manner, the inhibition of mAb binding to the gp41 by DT-P1 was significantly lower than that reported for the single E2 peptide. Thus, DT-P2 maintains, at the highest concentration, the recognition of the target of the original E2 peptide, whereas DT-P1 decreases its interaction capability with the disulfide loop region on gp41.

The target site of the E1 peptide fusion inhibitor, which is the HIV-1 FP (AVGIGALFLGFLGAAGSTMGARS), has also been studied for the DT-peptides. We have tested the recognition of both DT-peptides with the HIV-1 FP by means of the haemolysis assay. Although both DT-peptides were able to inhibit haemolysis induced by the FP to some extent at the highest concentrations (Fig. 2b), the inhibition pattern of DT-P2 was more similar to that previously reported for the E1 peptide fusion inhibitor^11^. The highest concentration tested of DT-P2 (5 µM) inhibited 70% whereas DT-P1 only inhibited 25% of haemolysis induced by FP.

**Figure 3:**
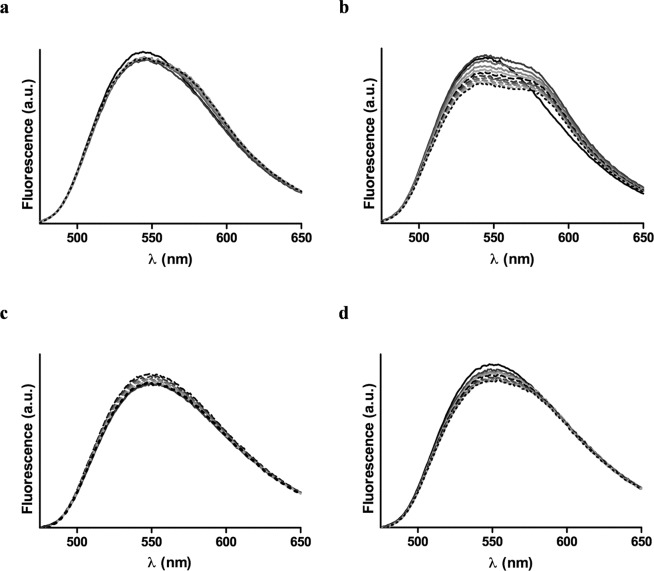
Fluorescence spectra of donor NBD-FP (0.5 µM) (black solid line) and acceptor labelled peptide mixtures in liposomes (100 uM) at concentrations ranging from 0.15 µM (grey solid line) to 1.5 µM (black dotted line). Acceptor labeled peptides: (**a**) TAMRA-DT-P1 in POPG liposomes; (b) TAMRA-DT-P2 in POPG liposomes; (**c**) TAMRA-DT-P1 in POPC liposomes; (**d**) TAMRA-DT-P2 in POPC liposomes.

To deep delve in the recognition of the FP as a target site by the DT-peptides, a Fluorescence Resonance Energy Transfer (FRET) assay was performed^22^. To this end, both DT-peptides were labeled on solid phase with 5(6)-carboxy-tetramethyl-rhodamine (TAMRA) at the N-terminus. The HIV-1 FP labeled with 6-(7-nitrobenzofurazan-4-ylamino) hexanoic acid (NBD) was used as a donor peptide. The fluorescence assay was assessed in a membranous environment using POPG liposomes as model membrane. As depicted in Fig. 3b, increased concentrations of TAMRA-DT-P2 resulted in a decrease of the maximal emission wavelength (λ_em_ = 544 nm) of NBD upon excitation at λ_ex_ = 467 nm, as well as an increase in the maximal emission of rhodamine (580 nm). These results demonstrated that there is an energy transfer from the donor-peptide to the acceptor-peptide due to the association between the HIV-1 FP and the DT-P2.

This energy transfer was considerably smaller when the FRET assay was carried out in POPC liposomes (Fig. 3d). As reported, HIV-1 FP shows an α-helical conformation in POPG vesicles at a 1:200 peptide:lipid ratio while adopts an extended structure in POPC membranes^23^. HIV-1 FP inserts deeply as an α-helix into the hydrophobic core of POPG membrane bilayer and lies on the surface of the lipid bilayer as extended conformation in POPC liposomes^24^. This different conformational behavior of HIV-1 FP depending on the membrane lipid composition, among other factors such as peptide to lipid molar ratio and concentration of divalent cations, has been related to its physiological activity^25^. Thus, the overall results of the FRET assays pointed out that the association between DT-P2 and FP is closer when the FP is embedded in the membrane in an α-helix conformation.

On the other hand, the fluorescence assay showed that the energy transfer between the NBD-FP and the TAMRA-DT-P1 was significantly lower in both POPG (Fig. 3a) and POPC (Fig. 3c) liposomes. Accordingly to the results of the haemolysis assay, the interaction of DT-P1 with the HIV-1 FP is minor and consequently, the DT-P1 is less able to maintain the target site of the original E1 peptide sequence.

Taking together the FRET results for DT-peptides and comparing their FRET efficiencies with the ones previously reported for the E1 peptide^11^, the association between FP and E1 peptide is significantly closer than that between FP and DT-P2. The energy transfer efficiency between FP and E1 peptide at a donor:acceptor 1:1 ratio, was around 30% while the energy transfer efficiency between FP and DT-P2 was only 5%.

The overall described biophysical assays demonstrated that DT-P2 maintains better the recognition of both gp41 target sites than DT-P1 but not as efficiently as the single E1 peptide. These results might explain the lower antiviral activity of DT-P1 and to some extent the loss of activity of DT-P2. To understand the lower efficiency of DT peptides in the recognition of the gp41 target sites and consequently their different functional behavior, conformational studies of the peptides on the membrane environment have been next carried out.

### DT-P2 resembles better the conformation of the E1 peptide in micelles

**Figure 4:**
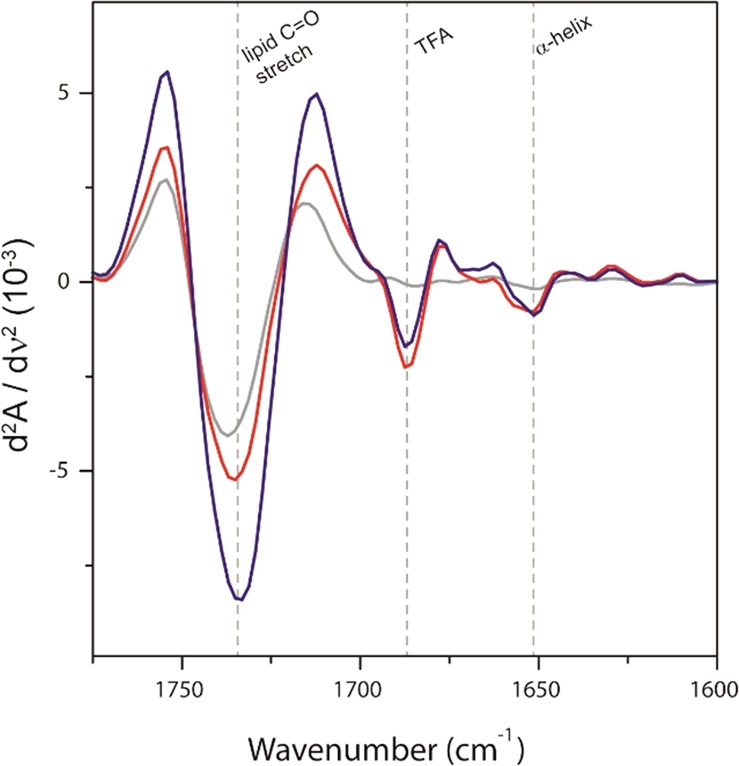
Absorbance second derivative of ATR-FTIR spectra for the C = O stretch and amide I regions for liposomes in the absence (grey line) and in presence of DT-P1 (red line) and DT-P2 (blue line) peptides in dry films. Since the peptide:lipid ratio (1:130) is prone to mask peptide secondary structures, we have used the second derivative of ATR-FTIR to highlight different secondary structures due to mathematical band narrowing. The intense peak at 1685 cm^−1^ indicates the presence of TFA, used as counter-ion in peptide synthesis. The dotted lines indicate the wavenumber position of regions of interest.

Previously reported computer-based structural analysis of the N-terminal part of the E2 protein demonstrated that the region that comprises the E2 peptide does not contain significant amounts of regular secondary structure and is rather flexible to interact with the disulfide loop of gp41 during HIV-1 entry^18^. By contrast, structure-activity studies carried out previously by our group have confirmed the importance of some structural elements in the anti-HIV-1 activity of the shorter inhibitor E1 peptide^19^. As previously determined in our group using NMR spectroscopy, E1 peptide is embedded and adopts a helix-turn-helix secondary structure in presence of DPC-d_38_ micelles. We decided to evaluate if the modification of E1 peptide in N- and C-terminal ends with E2-GGGS/GGGS-E2 sequences could affect the E1 main secondary structure. To assess qualitatively the structural changes, I-Tasser Server to predict secondary structure, conformational analysis by attenuated total reflection Fourier Transform Infrared spectroscopy (ATR-FTIR) in the presence of phosphatidylcholine (PC) vesicles and evaluation of 1D ^1^H NMR spectra of the DT-peptides in zwitterionic micelles were used^26^. First, the secondary structure prediction showed that in both peptides, the E1 moiety conserves the helix-coil-helix secondary structure (Supplementary Fig. S5). Second, in the presence of phosphatidylcholine (PC) the ATR-FTIR second derivative spectra indicated that both peptides show a characteristic α-helix peak position at 1650 cm^−1^ (Fig. 4).

**Figure 5:**
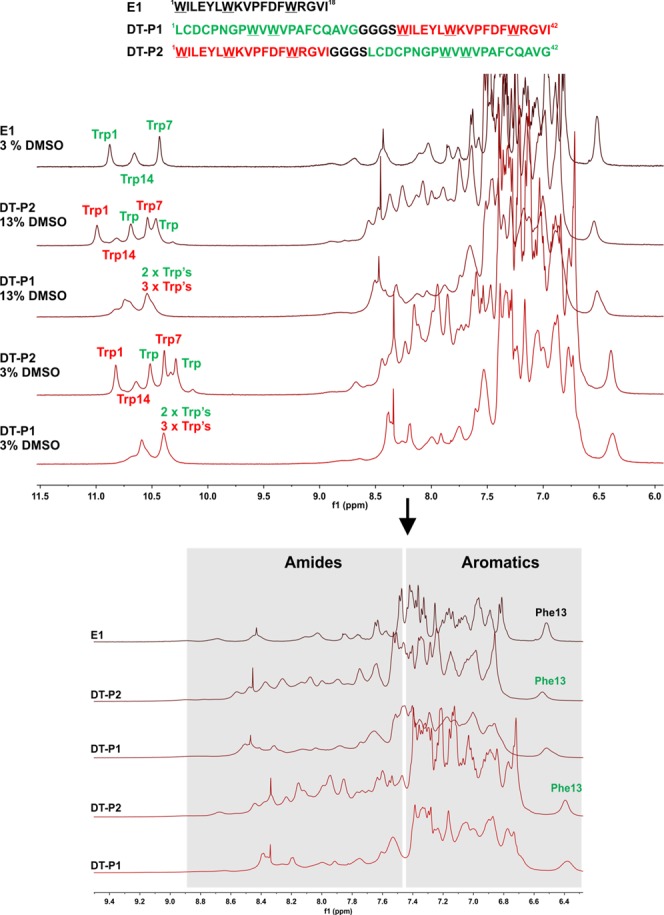
1D ^1^H NMR spectra of E1, DT-P1 and DT-P2 peptides in DPC micelles (15 mM HEPES-d_11_, pH = 6.0, 1 mM TCEP, in 87% H_2_O/13% DMSO-d_6_ or 87% H_2_O/10% D_2_O/3% DMSO-d_6_; 300 K). Phe-13 and Trp’s NH side chain assignment obtained from our previous work for E1^19^ and tentative assignment for DT-P2 peptide.

Third, regarding NMR the appearance of both tryptophan and aromatics regions in the DT-P2 peptide ^1^H spectrum in the presence of 50 mM DPC micelles (Fig. 5) was very similar from that for E1 peptide (Fig. 5). The E1 moiety in DT-P2 showed a similar chemical shift dispersion/pattern of tryptophan indole NH protons and Phe/Tyr aromatic protons, in particular Phe-13 aromatic protons (in E1P47 nomenclature) upfield shift when dissolved in presence of 50 mM DPC micelles^19,27^. Comparing the NMR spectra of peptides DT-P1 and DT-P2, the spectrum of the former peptide displayed more broadened resonances, as was observed for the loss of chemical shit dispersion for tryptophan side chain NH protons, thereby suggesting an increased aggregation state of this peptide in micelles. To increase solubility, higher amounts of DMSO-d_6_ were used in the final NMR sample (from 3 to 13%), but no improvement was observed.

To evaluate the aggregation state of both peptides, the translational diffusion coefficients were measured by NMR at low (0.5 mM) concentration for both peptides in 10 mM phosphate buffer (pH = 6.0, in 100% D_2_O: 0.5 mM DSS and 1.0 mM TCEP) with 50 mM DPC-d_38_ (Table 3). NMR measurements showed that the value of self-diffusion coefficients obtained for DT peptides is in the expected range for a peptide of similar length associated to micelles^19,28^. The diffusion coefficient of DT-P1 peptide decreases in comparison with the value measured for DT-P2. Taking into account that both peptides have the same molecular weight, the slower diffusion indicates that the radius/hydrodynamic volume of DT-P1 is bigger than DT-P2 and that DT-P1 is more aggregated in DPC micelles than DT-P2. Also, we observed a decrease in diffusion coefficients for DPC (*CH*_2_)_n_ and –*CH*_2_-N^+^(CH_3_)_3_ protons from DT-P2/DPC to DT-P1/DPC, which could be indicative of bigger micelles in presence of more aggregated DT-P1 peptide.

**Table 3:** Translational diffusion coefficient measured by NMR spectroscopy for peptide samples in DPC-d_38_ (10 mM phosphate buffer pH = 6 in 100% D_2_O, 1 mM TCEP and 0.5 mM DSS; 300 K).

	D (10^−11^ m^2^s^−1^)	error
**Sample DT-P1 + DPC**	**(0.50:50 mM)**	
DT-P1^*a*^	5.28	1.14 × 10^−14^
DPC^*b*^	8.24	7.96 × 10^−14^
DPC^*c*^	7.88	3.77 × 10^−14^
**Sample DT-P2 + DPC**	**(0.50:50 mM)**	
DT-P2^*a*^	6.94	7.64 × 10^−15^
DPC^*b*^	8.90	1.03 × 10^−14^
DPC^*c*^	8.93	**4.29 × 10** ^**−14**^

^a^Peptides Aromatic region 7.23–7.13 ppm,

^b^Two components: CH_2_ DPC region 1.26–1.14 ppm (small D, from free + micellar DPC) and small MW contaminant (large D, see fittings in supplementary material).

^c^CH_2_N^+^(CH_3_)_3_ DPC region 3.49–3.53 ppm.

In addition to the peptide conformation, it is relevant to carry out membrane affinity studies for the DT-peptides in order to understand their different functional behavior in relation to the E1 peptide.

### DT-peptides loose the capability of assembly into the model membrane demonstrated by the E1 peptide

**Figure 6:**
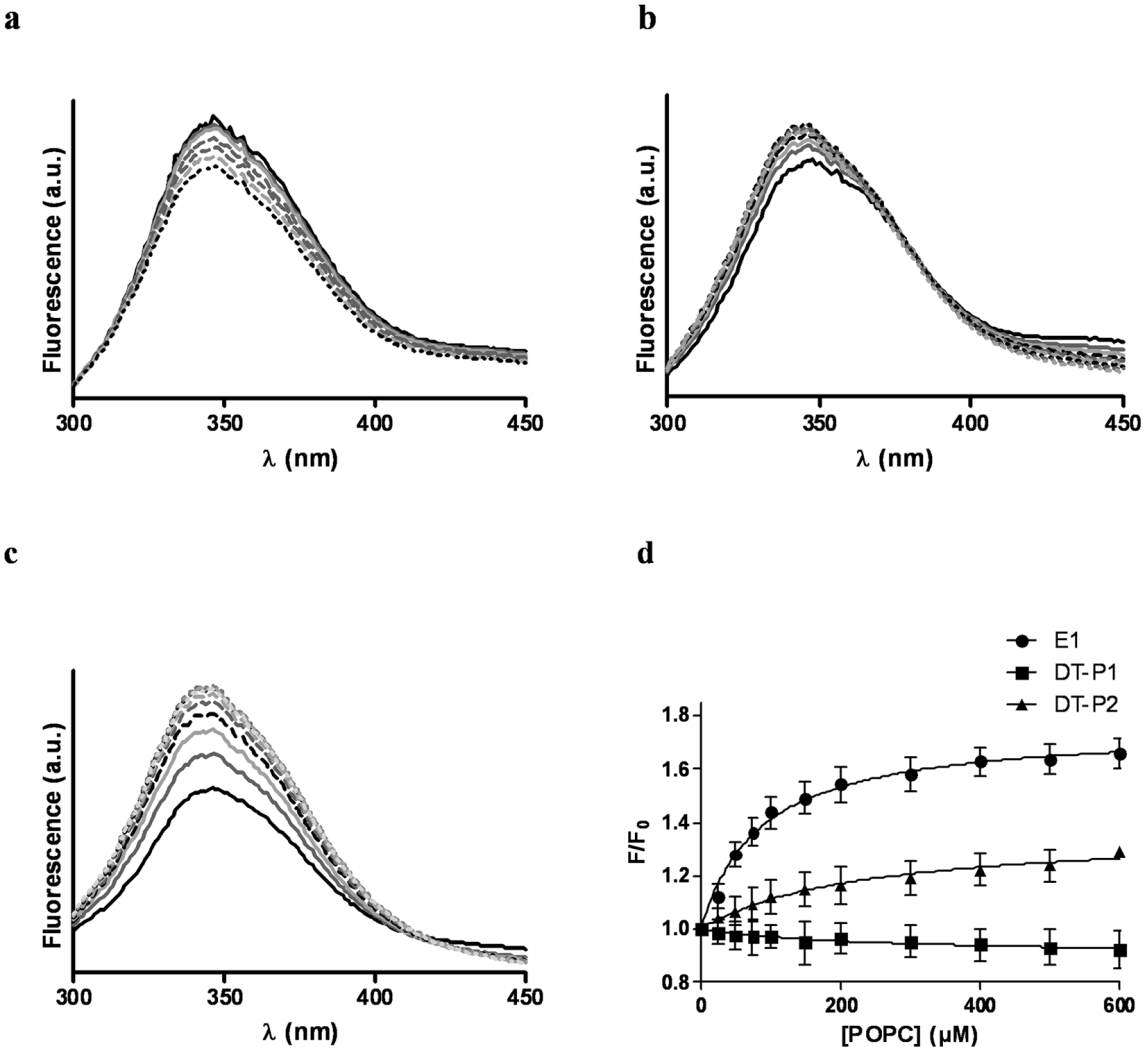
Fluorescence emission spectra of DT-P1 (**a**), DT-P2 (**b**) and E1 peptide (**c**) upon titration with POPC unilamellar vesicles. Black solid line represents 5 µM peptide in HEPES buffer; grey and dotted lines corresponds to peptide titration with POPC LUVs at a concentration ranged from 0.05 mM to 0.6 mM; (**d**) Partitioning isotherms of E1 and DT-peptides estimated from the fractional change in Trp fluorescence intensity upon addition of increasing amounts of liposomes. Binding experiments were done by triplicate. Values of apparent mole fraction partition coefficients were: K_X_ = 2.4 × 10^5^ and K_X_ = 8.2 × 10^5^ for DT-P2 and E1 peptide, respectively.

Membrane affinity was studied monitoring intrinsic fluorescence of tryptophan (Trp) residues after titration with unilamellar vesicles. Emission spectra of DT-peptides in buffer showed a maximum at a wavelength that was at least 10 nm blue-shifted related to the maximum emission of the soluble Trp derivative N-acetyltryptophanamide (NATA) indicating that Trp residues in both peptides are basically shielded from water. As illustrated in Fig. 6a, fluorescence intensity of Trp residues diminished after titration of DT-P1 with liposomes similarly as that of the Trp derivative (NATA) that was used to monitor the dilution effect after liposome titration. Unlike DT-P1, a fluorescence increase of the emission spectra of DT-P2 upon addition of POPC liposomes was observed (Fig. 6b). Nevertheless, the fluorescence increase of the Trp residues on the E1 peptide was higher at lower concentrations of lipid vesicles (Fig. 6c). The fractional change in peptide intrinsic fluorescence upon titration with liposomes represented in Fig. 6d demonstrated that although there is an interaction between DT-P2 peptide and the lipid bilayer that affects the environment of the Trp residues, the E1 peptide partitioned stronger into lipid vesicles.

**Figure 7:**
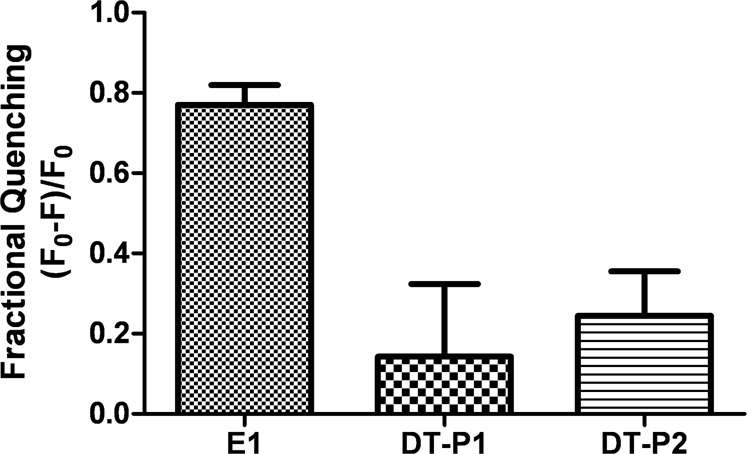
Peptide Trp quenching by brominated lipid vesicles. Fractional quenching is shown as mean ± SD of three repeated assays.

Collisional quenching of Trp by brominated phospholipids was used to assess the accessibility of the peptides to lipids. The quenching of Trp fluorescence by brominated lipids was examined after adding the peptides to lipid vesicles composed of 1,2-di-(9-10-dibromo)stearoyl-sn-glycero-3-phosphocholine and was evaluated by fluorescence comparison upon addition of the peptides to lipid vesicles composed of 1,2-dioleoyl-sn-glycero-3-phosphocholine. As shown in Fig. 7, both DT-peptides showed a limited accessibility for the lipids. On the contrary, Trp residues of the E1 peptide were highly quenched demonstrating greater peptide accessibility to the hydrophobic inner part of the lipid vesicles. This result is in agreement with previous structural NMR studies of the E1 inhibitor peptide in DPC micelles which indicated that it was lying parallel to the surface, with the negative-charged side chains oriented to the choline head group and the hydrophobic residues pointing to the micelle core^19^. Thus, the quenching of Trp residues of the E1 peptide by brominated atoms attached at positions 9 and 10 of the hydrophobic tails of the lipid implies that these Trp residues point to the micelle core such as it was previously reported. On the contrary, DT-peptides lose the capability of being soaked inside the lipid vesicles.

As it was previously demonstrated^19^, the interaction between the E1 fusion inhibitor and its viral target (FP) takes place at the membrane level. The intrinsic fluorescent experiments point out to the necessity that fusion inhibitor peptides that specifically interfere with the N-terminal region of gp41 are embedded within the membrane in order to interact properly with their viral target.

The conformational studies carried out in this work demonstrated that DT-P2, which has the E1 sequence on N-terminus, resembles better the helix-turn-helix structure of the E1 inhibitor and maintains better the recognition of both gp41 target sites. The reduced affinity of DT-P1 for the gp41 target sites could be related to its tendency to self-aggregate such as it was demonstrated by the translational diffusion coefficients measured by NMR. In order to reduce the peptide aggregation, longer and more hydrophilic linkers could be used to separate E1 and E2 sequences on novel modified DT- peptides. Taking into account that the two target regions are separated by the NHR in gp41, polyethylene glycol-based spacers of different lengths could also facilitate the simultaneous bind of DT-peptides to both targets. However, since the fusion process is progressive it is possible that there is no need to fix the distance between the two inhibitory domains.

Although DT-P2 is less prone to aggregation in micelles and preserve better the conformational features required for maintaining the antiviral activity than DT-P1, similar inhibition activities in both DT-peptides were found. Regarding the antiviral activity of the single E1 peptide, the lower inhibitory activity of both DT-peptides could be related with their lower interaction and assembly into the membrane phospholipids which impairs the recognition of the gp41 target site.

## Conclusions

The study of the functional behavior of DT-peptides in a membranous environment attending to the peptide recognition of the target sites on gp41, the peptide conformation as well as the peptide affinity to the membrane, lead us to conclude that the lower antiviral activity of the DT-inhibitors compared to single peptides could be related to the peptide affinity and its subsequent assembly into the model membrane. Specifically, the insertion of the peptide into the lipid bilayer is a necessary condition for maintaining the recognition of the target sites, mainly the fusion peptide on gp41, and therefore is closely related to the antiviral activity. The overall results point out to the requirement that fusion inhibitor peptides that interfere with those domains in HIV-1, critical for stabilizing the six-helix bundle formation, are embedded into membranes in order to interact with their viral target.

## Methods

The work was approved by the Ethical Committee of the Consejo Superior de Investigaciones Científicas (CSIC), Madrid, Spain. All methods were performed in accordance with the relevant guidelines and regulations of IQAC-CSIC.

### Synthesis, characterization and purification of peptides

The dual-targeting-peptides were manually synthesized by solid-phase peptide synthesis (SPPS) using 9-fluorenyl-methoxycarbonyl (Fmoc) strategy. DT-P1 and DT-P2 were synthesized as C- terminal carboxamides. Elongation of both peptidyl-resins was carried out following the same procedure. Thus, couplings were performed by 2-(1H-7-azabenzotriazole-1-yl)-1,1,3,3-tetramethyluronium hexafluorophosphate methanaminium (HATU) and diisopropylethylamine (DIPEA) activation, with a threefold molar excess of Fmoc-amino acids. Amino acid side chain protection was effected by the following: triphenylmethyl (Trt) for glutamine, asparagine and cysteine; tert-butyl (tBu) for aspartic acid, glutamic acid, serine and tyrosine; 2,2,5,7,8-pentamethyl-chroman-6-sulfonyl (Pmc) for arginine; and tert-butoxycarbonyl (Boc) for lysine and tryptophan. The Fmoc deprotection step was performed twice with 20% piperidine in dimethylformamide (DMF) for 10 min. The stepwise addition of each residue was assessed by Kaiser’s (ninhydrin) test and by the chloranil test for identification of secondary amines, when coupling proline residues.

A fraction of the DT-P1 and DT-P2 peptidyl-resins were labelled at the N-terminus with 5(6)-carboxy-tetramethyl-rhodamine (TAMRA), previously activated with N,N´-diisopropylcarbodiimide (DIPCDI) and 1-hydroxybenzotriazole (HOBt). The coupling reaction was carried out overnight with a threefold excess of the reagents.

The peptides were concomitantly side chain deprotected and cleaved from the resin by treatment with a mixture of 94% (v/v) trifluroacetic acid (TFA), 1% (v/v) triisopropylsilane (TIS), 2.5% (v/v) 2-mercaptoethanol and 2.5% (v/v) water for 3 h with occasional agitation at room temperature. The solvent was removed in vacuum and the crude peptides were precipitated with diethyl ether. The solids were dissolved in 30% acetic acid in water and lyophilized.

The crude peptides were purified by semipreparative HPLC in an XBridgeTM Prep C8 column (5 μm, 10 × 250 mm, Waters). The identity of the peptides was confirmed by electrospray ionization mass spectrometry (ES-MS). ES-MS was performed with a liquid chromatograph–time of flight (LC-TOF) detector, LCT Premier XE (Micromass Waters, Milford, MA, USA), coupled to Analytical Ultra Performance Liquid Chromatography apparatus (UPLC, Waters). Samples were dissolved in a mixture of acetonitrile/water (1/1, v/v) and analysed in the UPLC at a flow rate of 0.3 mL/min. The mass spectra were recorded in positive ion mode in the m/z 500–2500 range. UPLC was performed in an Acquity UPLC BEH C18 reverse-phase column (2.1 × 100 mm, 1.7 μm particle size) and with an Acquity UPLC (Waters) chromatograph. Solvent A was 20 mM formic acid in water and solvent B was 20 mM formic acid in acetonitrile. Elution was performed with linear gradients of 5–100% solvent B into A over 10 min at a flow rate of 0.3 mL/min. Characterization of the pure peptides ES-MS are shown in Supplementary Figs S1–S4. The peptides were 95% pure by analytical HPLC at 220 nm.

### Competitive ELISA assays

Nunc MaxiSorp 96-microwell plates (Nunc, Roskilde, Denmark) were used as a support for coating a solution of 0.22 ug/ml of HIV-1 gp41MN protein in carbonate/bicarbonate buffer (0.1 M, pH 9.8) overnight at 4 °C. After blocking the wells with 1% (w/v) BSA in phosphate buffer, pH 7.2 with 0.1% Tween 20 for 1 h at room temperature, peptides diluted in phosphate buffer were added to each well at graded concentration. Simultaneously, a solution of F240 monoclonal antibody in phosphate buffer was added to the wells at a 0.04 µg/well concentration. The plates were incubated for one hour and a half at room temperature. After washing three times, anti-human IgG conjugated to peroxidase (Dako Denmark) diluted 1:6000 in phosphate buffer was incubated for 1 h at room temperature. Then, the o-phenylenediamine dihydrochloride was incubated for 30 min and the absorbance values were measured at a wavelength of 492 nm. The measurements were taken by triplicate in three different days. The results are expressed as the percentage of inhibition of gp41 binding induced by the peptide.

### Haemolysis assay

The human care of the rabbits used to perform the haemolysis assay with blood samples was carried out according to the guidelines for animal care in the facilities of the IQAC-CSIC.

The procedure of the haemolysis assay was performed as described in ref. ^11^. Briefly, concentration solutions of 1.25, 2.5 and 5 µM of each DT- peptide and 100 µM of HIV-1 FP were incubated for 60 min at 37 °C. Erythrocytes from rabbit, previously washed with PBS and diluted 1:10 in PBS, were added at a concentration of 10%(v/v) to the peptide solutions and incubated again 60 min at 37 °C. The solutions were centrifuged at 1000 g for 5 min and supernatants were transferred to a 96 well Maxisorp^TM^ plate (Nunc). Absorbance was quantified at 405 nm. The percentage of haemolysis is expressed relative to the total haemolysis caused by the respective HIV-1 FP alone and is calculated as described in ref. ^29^. Each sample was processed in triplicate and on two different days. Haemolysis induced by each DT-peptide incubated with red blood cells without HIV-1 FP was also tested.

### Fluorescence assays

Fluorescence assays were performed in a PTI Fluorescence Master Systems spectrofluorimeter (Photon Technology International) using a 1 cm path length quartz cuvette.

Unilamellar vesicles (LUV) composed of the negatively charged phospholipid 1-palmitoyl-2-oleoyl-sn-glycero-3-phospho-(1′-rac-glycerol) (POPG) or zwitteronic phospholipid palmitoyl-2-oleoyl-sn-glycero-3-phosphocholine (POPC) (both from Avanti Polar-Lipids) were prepared as model membranes. Lipids (10 mM) were dissolved in chloroform/methanol (2/1, v/v) solution. The solvent was evaporated under reduced pressure. A buffer solution of PBS 0.01 M pH 7.4 was added to the previously obtained lipid film to prompt the spontaneous formation of multilamellar vesicles that were subjected to a process of 10 freeze and thaw cycles. The liposomes were then subjected to an extrusion process through 100-nm pore-sized polycarbonate filters in a high-pressure extruder.

To perform fluorescence resonance energy transfer assays, NBD-labelled FP and TAMRA-labelled DT-peptides were used as donor and acceptor, respectively. The emission spectra were recorded in the wavelength range 450–650 nm upon excitation at λ = 467 nm. NBD FP was added to a 100 µM suspension of LUVs to reach a final concentration of 0.5 µM. Then, TAMRA-labelled DT-peptides in doses ranging from 0.15 µM to 1.5 µM were sequentially added to the LUV solutions containing the NBD-FP. Fluorescent measurements were recorded before and after de addition of TAMRA-labelled peptides and intensities were corrected by subtraction of the signal produced by the labelled acceptor alone. The contribution of the vesicles was subtracted from every measurement. The percentage efficiency of the energy transfer was determined from the ratio of the donor in the presence and the absence of the acceptor, at the wavelength of the maximal emission of the donor (λ = 530 nm)^30^.

To carry out membrane partition studies with DT-peptides, emission fluorescence spectra were recorded for DT-peptides in PBS (0.01 M, pH 7.4) at 20 °C using an excitation wavelength of 280 nm. Changes in the tryptophan (Trp) fluorescence spectra were monitored after incubation of 5 µM of each peptide with increasing concentrations of liposomes. The suspensions were continuously stirred and were left to equilibrate for 10 min before recording the spectra. Fluorescence intensities were corrected by subtraction of the vesicle blank. A lipid titration of N-acetyltryptophanamide (NATA) was carried out at the same time. As described in ref. ^31^, the apparent mole fraction partition coefficients were determined by fitting the binding curves to the equation:

$$F={f}_{bound}{F}_{max}+(1-{f}_{bound}){F}_{0}$$

where *F* is the relative fluorescence intensity, *F*_0_ is the fluorescence intensity in the absence of lipid.

$${f}_{bound}={K}_{x}L/(W+{K}_{x}L)$$

where *K*_*x*_ is the mole fraction partition coefficient, *L* is the lipid concentration, and *W* is the molar concentration of water (55.3 M at 25 °C).

Quenching of Trp by brominated phospholipid was performed using liposomes composed of 1,2-di-(9-10-dibromo) stearoyl-sn-glycero-3-phosphocoline (Br-PC) (Avanti Polar Lipids). As previously reported^32^, this lipid has similar properties as unsaturated phosphatidylcholine chains due to the bulkiness of bromine residues. For this reason, liposomes of 1,2-dioleoyl-sn-glycero-3-phosphocholine (DOPC) were used for comparing the Trp fluorescence of peptides with brominated liposomes. Liposomes were prepared following the same procedure as described above. Comparatively, a concentration of 2 µM of peptides was added to 200 µM of lipid vesicles either BrPC or DOPC. After 5 min of incubation at 25 °C, fluorescence spectra were recorded with an excitation wavelength of 290 nm and emission from 300 to 450 nm. The fractional quenching was calculated as reported in ref. ^33^ by *(F*_0_ − *F)/F*_0_, where *F*_0_ is the fluorescence intensity at 340 nm for peptides added to DOPC liposomes and *F* for peptides added to Br-PC liposomes.

For fluorescein leakage experiments, liposomes of soy bean phosphatidylcholine (PC) were prepared containing 60 mM fluorescein. At this concentration the dye exhibits autoquenching. Multilamellar liposomes, obtained after hydrating the lipid film with phosphate buffer 10 mM pH 7.4 containing 60 mM fluorescein, were extruded through 100 nm pore polycarbonate membranes. Non-entrapped fluorescein was removed by size exclusion chromatography using PD-10 columns with Sephadex G-25 (medium), eluted with phosphate buffer 10 mM pH 7.4 containing 60 mM NaCl. Typically, a solution of purified liposomes of about 0.65 mM PC was obtained.

The effect of the peptides on the PC membranes was monitored by measuring the fluorescence of the purified liposomes after the addition of aliquots of DMSO solutions of peptides to a final peptide concentration of 2.5 µM (final DMSO content was 10% v/v). Since the addition of the peptides caused vesicle disruption, a scattering contribution to the very fluorescence signal was produced. Thus fluorescence could not be accurately measured for more than, approximately, 50 minutes. After incubation, Triton X-100 was added to the samples in order to solubilize any lipidic structure and to measure their total fluorescence. Results are expressed as % of entrapment using the following equation:

$$ \% \,{\rm{entrapment}}=100\,(1\,\mbox{--}\,({F}_{i}\,\mbox{--}\,{F}_{0})/({F}_{f}\,\mbox{--}\,{F}_{0}))$$

where *F*_0_, *F*_*f*_ and *F*_*i*_ are, respectively, the sample fluorescence at time 0, after Triton X-100 addition, and at time *i*.

### Attenuated total reflection Fourier Transform Infrared (ATR-FTIR) experiments

10 µL of fluorescein-free suspensions containing peptide mixed with liposomes (1:130) placed on top of the ATR ZnSe crystal, were gently dried under a N_2_ stream. Spectra were obtained at room temperature by the average of 2 blocks of 250 scans each at 2 cm^−1^ resolution in a VARIAN FTS-7000 infrared spectrometer. The second derivative was performed with nine smoothing points.

### NMR Spectroscopy

Nuclear magnetic resonance (NMR) spectra were recorded at 300 K on a Bruker Avance III console (Bruker Biospin, Germany) combined with a 9.7 T Oxford Instruments magnet (^1^H 500 MHz), equipped with a 5 mm inverse cryogenically cooled triple-resonance TCI (^1^H, ^13^C, ^15^N, and ^2^H lock) cryoprobe with a z-axis magnetic field-gradient capability. For proton spectra acquisition, peptide samples were prepared by dissolving first lyophilized peptide in a small amount of DMSO-d6 and next, dissolution in detergent solution containing perdeuterated DPC-d_38_ (final molar ratio ~ 1:100, peptide/DPC 0.5:50 mM). The final pH of peptide/detergent aqueous solution (87% H_2_0/10% D_2_0/3% DMSO-d6 or 87% H_2_0/13% DMSO-d6, 15 mM HEPES-d_11_) was adjusted to 6.2. Buffer solution included 1 mM of reducing agent Tris(2-carboxyethyl)phosphine hydrochloride (TCEP), to minimize aggregation by intermolecular disulfide bond formation. For diffusion coefficients determination, the field gradient strength (Gz) was calibrated by measuring the self-diffusion coefficient of residual H_2_O in a 100% ^2^H_2_O sample at 298.0 K. A diffusion coefficient of 1.91 × 10^−9^ m^2^ s^−1^ for the residual H_2_O signal was then used for the back calculation of the field gradient strength. Diffusion NMR experiments were acquired at 300 K to minimize convection effects. The stebpgp1s19 pulse sequence with WATERGATE 3919 for water suppression and one spoil gradient from Bruker library was used. The diffusion coefficient, D, was determined by fitting diffusion weighted intensities of selected peaks or integrals over a chosen range to the following equation

1 $${I=I}_{{\rm{0}}}\exp \{\,-\,{\gamma }^{{\rm{2}}}{{\rm{g}}}^{{\rm{2}}}{{\rm{\delta }}}^{{\rm{2}}}({\rm{\Delta }}-{\rm{\delta }}/3)D\},$$

where γ is the gyromagnetic ratio of proton and g, δ and Δ are the amplitude, duration and separation of the single pair of gradient pulses, respectively. Due to the use of bipolar gradient elements, Δ is replaced by (Δ − τ_1_/2) in Eq. (1), with τ_1_ being the time interval between the bipolar gradient pulses within the bipolar gradient encoding or decoding segment. Fittings to Eq. (1) were performed using Bruker Dynamics Center software (Bruker Biospin, Germany), the results are reported below. In the case of DPC, the observed diffusion coefficient is the weighted average of the free (*D*_free_) and bound (*D*_bound_) values and

2 $${D}_{{\rm{obs}}}={f}_{{\rm{free}}}{D}_{{\rm{free}}}+{f}_{{\rm{bound}}}{D}_{{\rm{bound}}},$$

in which *f*_free_ and *f*_bound_ are the mole fraction of free and micellar DPC. In our case, D_bound_ (DPC) = D_mic_ (DPC) ≅ D_mic_ (peptides). The reason for DPC diffusion coefficients being higher than peptides values is likely due to a surplus of free monomeric DPC molecules (below cmc), so the diffusion coefficient measured for DPC has a relevant contribution of DPC-free peptide, while the observed peptide resonances (and their diffusion coefficient) mainly correspond to DPC-bound peptide.

### Virus preparation and titration

The preparation of the HIV-1 Env pseudotyped virus stocks in a GCLP- (Good Clinical Laboratory Practice) compliant manner occurred as described previously^34^. This includes the pseudoviruses listed in Table 2 with the Env sequences HXB2, AC10.0.29, Q461.e2, Q23.17, CAP210.2.00.E8 and CM235.c11 as well as the Env deficient backbone plasmid pSG3ΔEnv. Within this study the titration assay to determine the virus dilution at a relative luminescence unit (RLU) of 150,000 was performed according to the method described before^34–36^ and the luminescence was measured using the Infinite F200 microplate reader.

### TZM-bl Assay

The neutralization assay utilizing the TZM-bl cells was conducted to analyze lot-to-lot variations of freshly prepared virus stocks as published by Montefiori in 2009^36,37^. This is a modified version of the assay by Wei *et al*.^38^ and determine the Inhibitory Concentration providing 50 value (IC_50_ = 50% reduction of the RLU values compared to the virus control; half maximal inhibitory concentration) by measuring the luminescence with the Infinite F200 microplate reader. Within the assay a panel of five specified control reagents was assayed: sCD4 (Progenics Pharmaceutical, Tarrytown, USA), IgG1b12, 2F5, 4E10 and TriMab consisting of 2G12, IgG1b12 and 2F5 (Polymun Scientific, Klosterneuburg, Austria) with an initial concentration of 25 µg/ml. The indicated concentration of the control reagents, which should lead to 50% neutralization, must agree within 3-fold between the newly prepared virus stock and the reference virus for 80% of the tested reagents.

### Cell viability and antiretroviral assays

Peptides were tested for their antiviral activities against a panel of HIV pseudotypes carrying different HIV envelope proteins (Table 2) in the TZM-bl indicator cell line. Briefly, 10-fold serial dilutions of the compounds (0.001–10 µM) were incubated in duplicates with the cells in 96-well plates. 1 h after incubation, cell culture medium was replaced and cells were infected with the different pseudotypes together with fresh compounds. TZM-bl cells were infected with a virus inoculum corresponding to relative light units (RLUs) of 10-fold higher than the background RLUs in control cells. 48 h after infection, cells were washed and luciferase was quantified using the Britelite Plus Luciferase Assay System (PerkinElmer). Luciferase values were normalized to DMSO control. Cell viability was estimated in parallel in non-infected TZM-bl cells with the Cell Titer Glo ATPase kit (Promega). CC_50_ and EC_50_ values ± SEM were calculated with GraphPad Prism, using the built-in function log (inhibitor) vs. normalized response.

## Supplementary information

PEPTIDE ASSEMBLY ON THE MEMBRANE DETERMINES THE HIV-1 INHIBITORY ACTIVITY OF DUAL-TARGETING FUSION INHIBITOR PEPTIDES
