# Supplementary material for: Peptide Assembly on the Membrane Determines the HIV-1 Inhibitory Activity of Dual-Targeting Fusion Inhibitor Peptides
Source: Sci Rep. 2019 Mar 1;9:3257. doi: 10.1038/s41598-019-40125-4 (PMC6397244; doi:10.1038/s41598-019-40125-4)
Supplement: Supplementary file 1 — PEPTIDE ASSEMBLY ON THE MEMBRANE DETERMINES THE HIV-1 INHIBITORY ACTIVITY OF DUAL-TARGETING FUSION INHIBITOR PEPTIDES [file 41598_2019_40125_MOESM1_ESM.docx]

**SUPPORTING INFORMATION**

**PEPTIDE ASSEMBLY ON THE MEMBRANE DETERMINES THE HIV-1 INHIBITORY ACTIVITY OF DUAL-TARGETING FUSION INHIBITOR PEPTIDES**

Maria J. Gomara^a*^, Yolanda Pérez^b^, Javier P. Martinez^c^, Ramon Barnadas-Rodriguez^e^, Anke Schultz^f^, Hagen von Briesen^f^, Alex Peralvarez-Marin^e^, Andreas Meyerhans^c,d^ and Isabel Haro^a^*.

**Table of Contents:**

Figure S1. Electrospray mass spectrometry (ES-MS) of the DT-P1 peptide

Figure S2. ES-MS of the DT-P2 peptide

Figure S3. ES-MS of the TAMRA-DT-P1

Figure S4. ES-MS of the TAMRA-DT-P2

Figure S5. Secondary structure prediction for DT-peptides using I-Tasser server

Figure S6. Dynamics Center Properties of D fit of DT-P1 sample

Figure S7. Dynamics Center Properties of D fit of DT-P2 sample

**DT-P1**


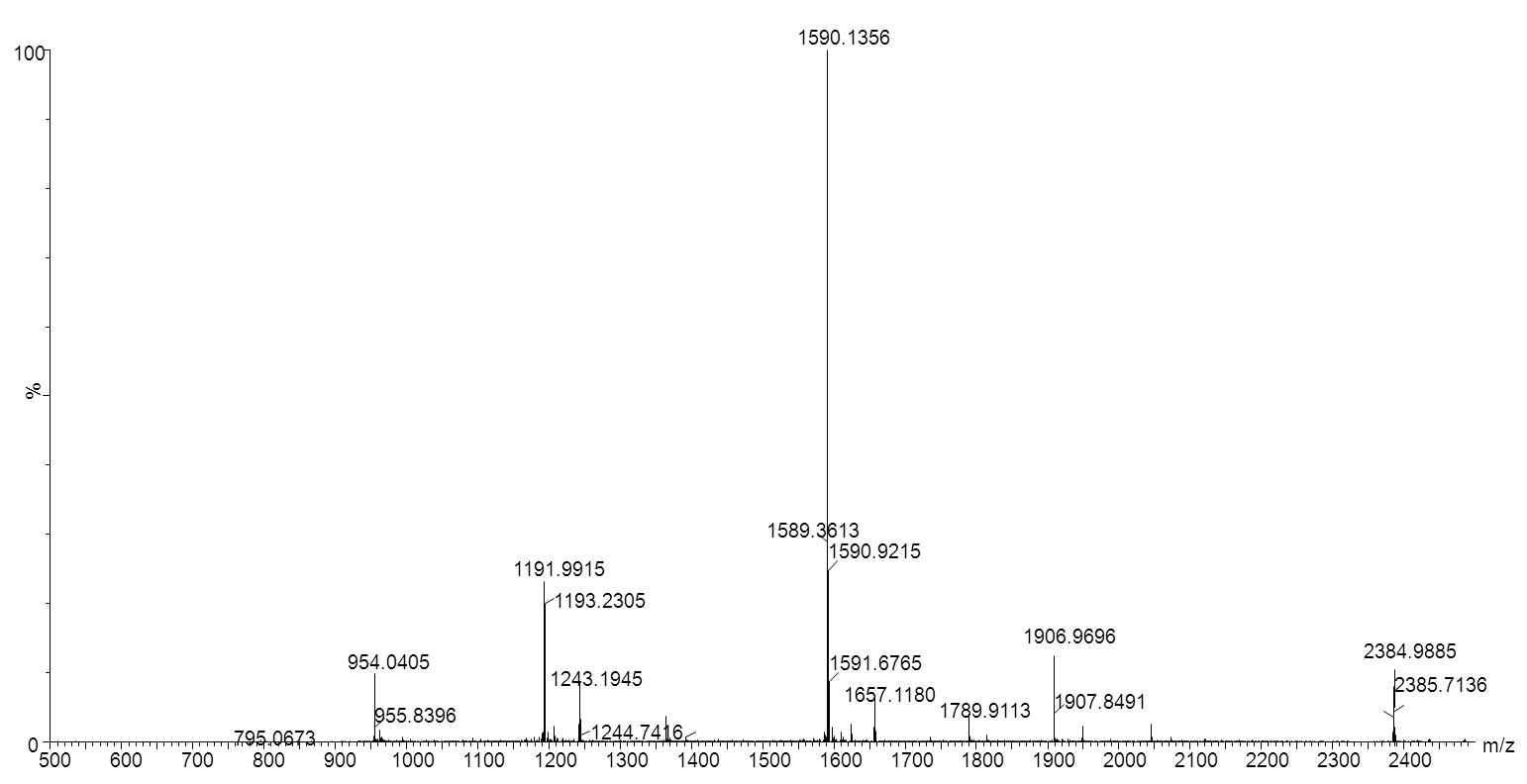


**Figure S1**. MS characterization of DT-P1 peptide. Calculated m/z: [M+2H]^+2^=2384.17, [M+3H]^+3^=1589.78, [M+4H]^+4^=1192.59; experimental m/z: [M+2H]^+2^= 2384.99, [M+3H]^+3^= 1590.14, [M+4H]^+4^=1192.0

**DT-P2**


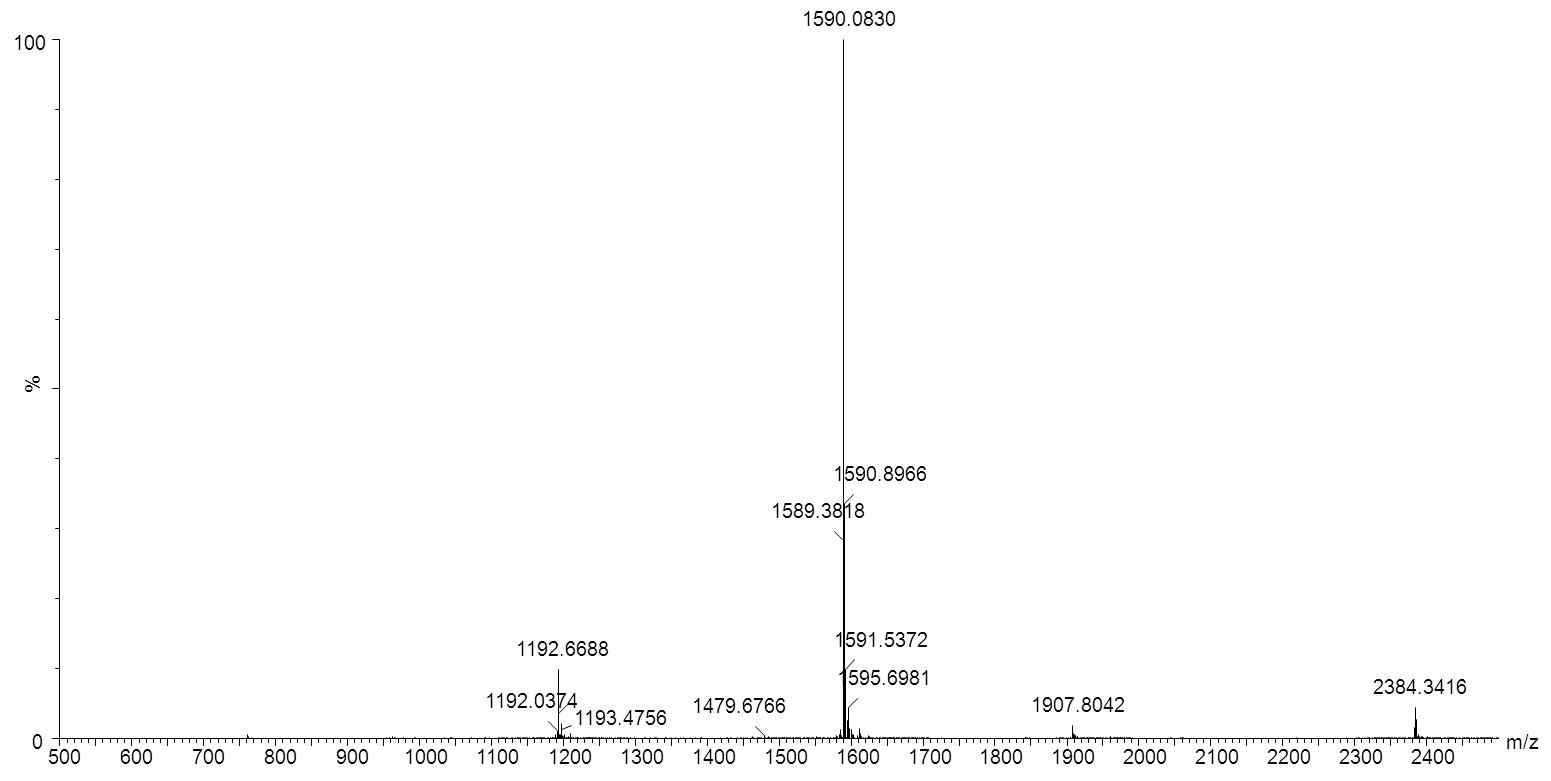


**Figure S2**. MS characterization of DT-P1 peptide. Calculated m/z: [M+2H]^+2^=2384.17, [M+3H]^+3^=1589.78, [M+4H]^+4^=1192.59; experimental m/z: [M+2H]^+2^= 2384.34, [M+3H]^+3^= 1590.08, [M+4H]^+4^=1192.67

**TAMRA-DT-P1**


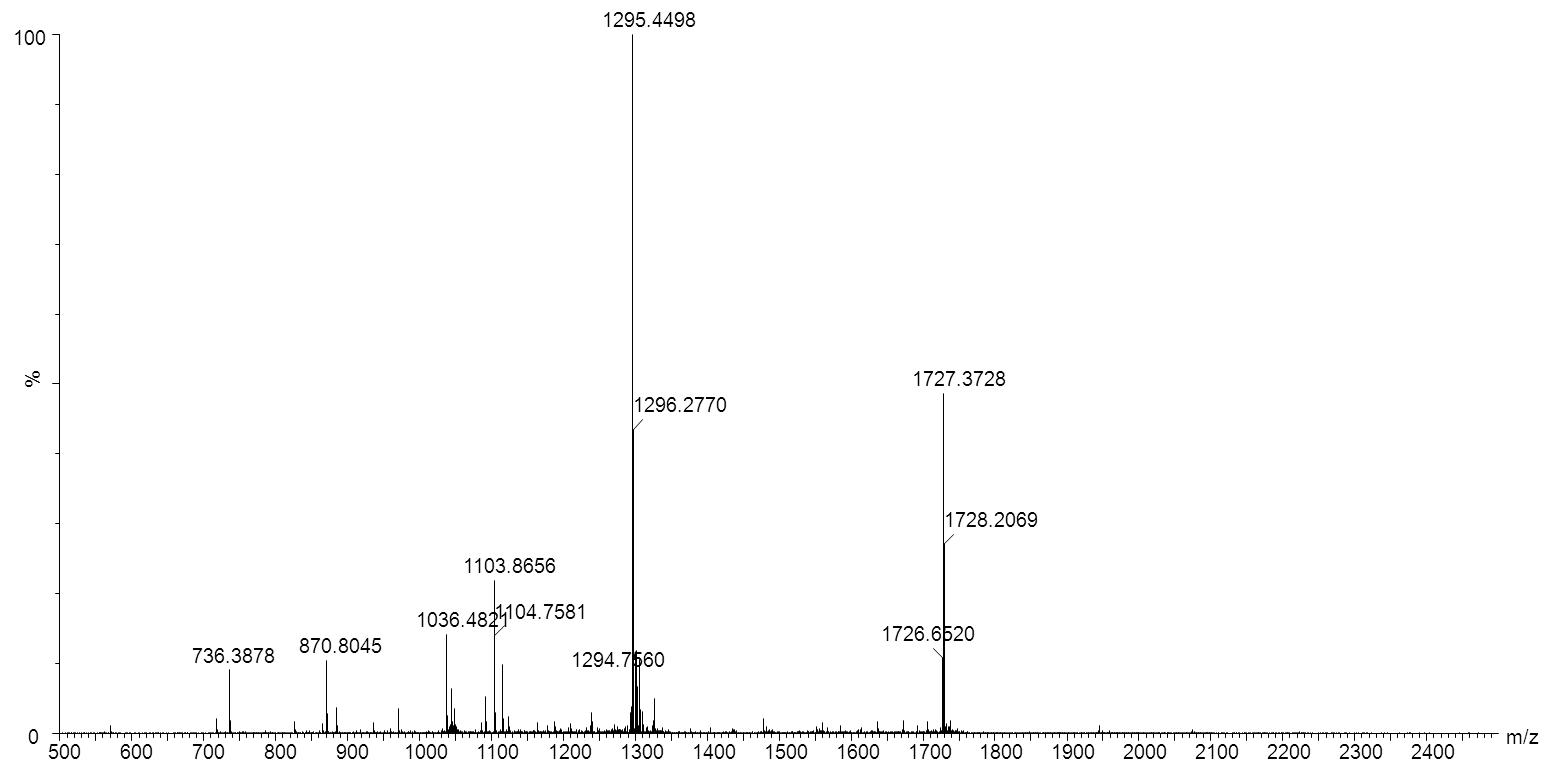


**Figure S3**. MS characterization of TAMRA-DT-P1 peptide. Calculated m/z: [M+3H]^+3^=1727.16, [M+4H]^+4^=1295.62; experimental m/z: [M+3H]^+3^= 1727.37, [M+4H]^+4^=1295.45

**TAMRA-DT-P2**


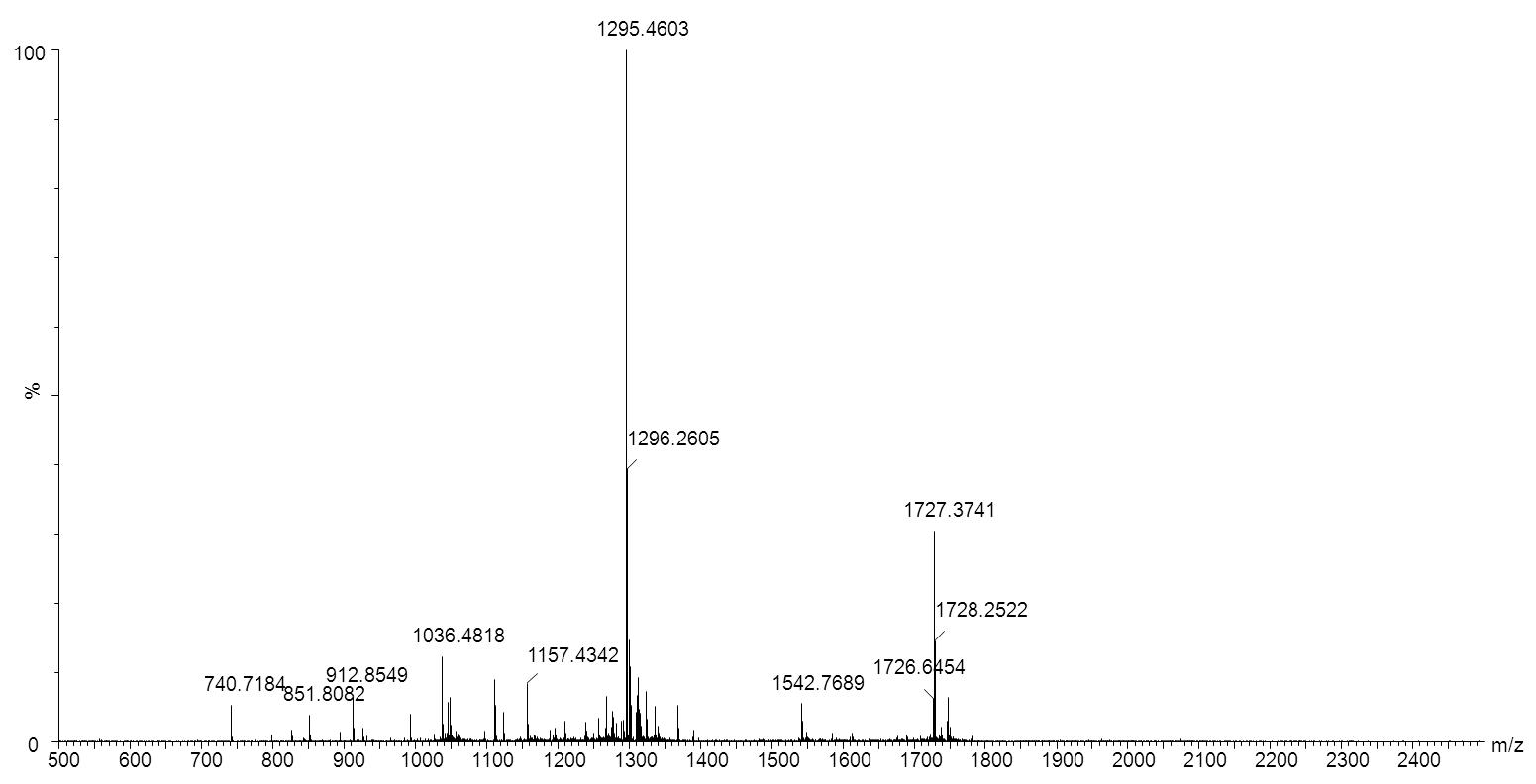


**Figure S4.** MS characterization of TAMRA-DT-P2 peptide. Calculated m/z: [M+3H]^+3^=1727.16, [M+4H]^+4^=1295.62; experimental m/z: [M+3H]^+3^= 1727.37, [M+4H]^+4^=1295.46

| Sequence | WILEYLWKVPFDFWRGVIGGGSLCDCPNGPWVWVPAFCQAVG |
| --- | --- |
| Predicted 2ary structure | **CHHHHHHHCCHHHHHCCCCCCCCCCCCCCCSSSHHHHHHHCC** |
| Confidence score  2ary structure | **968887738706774200575304699998684489775549** |
| Predicted Solvent Accessibility | **433433242334313322443321414723221013315438** |

| Sequence | LCDCPNGPWVWVPAFCQAVGGGGSWILEYLWKVPFDFWRGVI |
| --- | --- |
| Predicted 2ary structure | **CCCCCCCCSSSHHHHHHHHCCCCHHHHHHHHHCCHHHHHCCC** |
| Confidence score  2ary structure | **987989986744899988368615799999848705652349** |
| Predicted Solvent Accessibility | **727147332220133043334423210321242334324437** |

**Figure S5**. Secondary structure prediction for DTP peptides using I-Tasser server.^3^ Predicted secondary structure: H (helix), S (Strand) and C (Coil). Predicted solvent accessibility: values range from 0 (buried residue) to 9 (highly exposed residue).

**Figure S6**. Dynamics Center Properties of D fit of DT-P1 sample

DT-P1 sample: 0.5 mM DT-P1 in 10 mM phosphate buffer (pH = 6.0, in 100 % D_2_O: 0.5 mM DSS and 1.0 mM TCEP) with 50 mM DPC-d_38_.

1. **DT-P1 Aromatic Region (6.68-7.46 ppm)**

results of peak 1

ppm 7.068

fitted function f (x) = Io * exp (-D * x^2 * gamma^2 * littleDelta^2 (bigDelta-littleDelta/3)* 10^4

calculated Io 2.023e+10 error 1.161e+06

calculated D 5.281e-11 error 1.141e-14 m2/s

used gamma: 26752 rad/(s*Gauss)

used little delta: 0.0020000 s

used big delta: 0.14990 s

used gradient strength: variable

x variables all variables used even if identical

fit points all non-zero integrals/volumes used

numerical values

X variable [G/cm] original Y fitted Y residual

1.1830 2.0014e+10 2.0225e+10 -2.1124e+08

4.1700 1.9876e+10 2.0152e+10 -2.7584e+08

7.1580 1.9786e+10 1.9999e+10 -2.1236e+08

10.145 1.9674e+10 1.9767e+10 -9.2924e+07

13.133 1.9432e+10 1.9459e+10 -2.6919e+07

16.121 1.9133e+10 1.9079e+10 5.3602e+07

19.108 1.8758e+10 1.8631e+10 1.2685e+08

22.096 1.8299e+10 1.8121e+10 1.7808e+08

25.084 1.7758e+10 1.7554e+10 2.0397e+08

28.071 1.7143e+10 1.6936e+10 2.0730e+08

31.059 1.6452e+10 1.6274e+10 1.7768e+08

34.046 1.5718e+10 1.5576e+10 1.4197e+08

37.034 1.4951e+10 1.4847e+10 1.0436e+08

40.022 1.4182e+10 1.4095e+10 8.6408e+07

43.009 1.3394e+10 1.3328e+10 6.6079e+07

45.997 1.2559e+10 1.2552e+10 6.6607e+06

48.984 1.1719e+10 1.1774e+10 -5.4220e+07

51.972 1.0877e+10 1.0999e+10 -1.2196e+08

54.960 1.0047e+10 1.0234e+10 -1.8739e+08

57.947 9.2354e+09 9.4841e+09 -2.4870e+08

Goodness of fit:

SSE (final chi) 209236.7273

R-square 0.9979

R-square adjusted 0.9978

RMSE 107.8159

2-sided ShapiroWilk test 0.2178

Interpretation Normal distribution of residuals likely


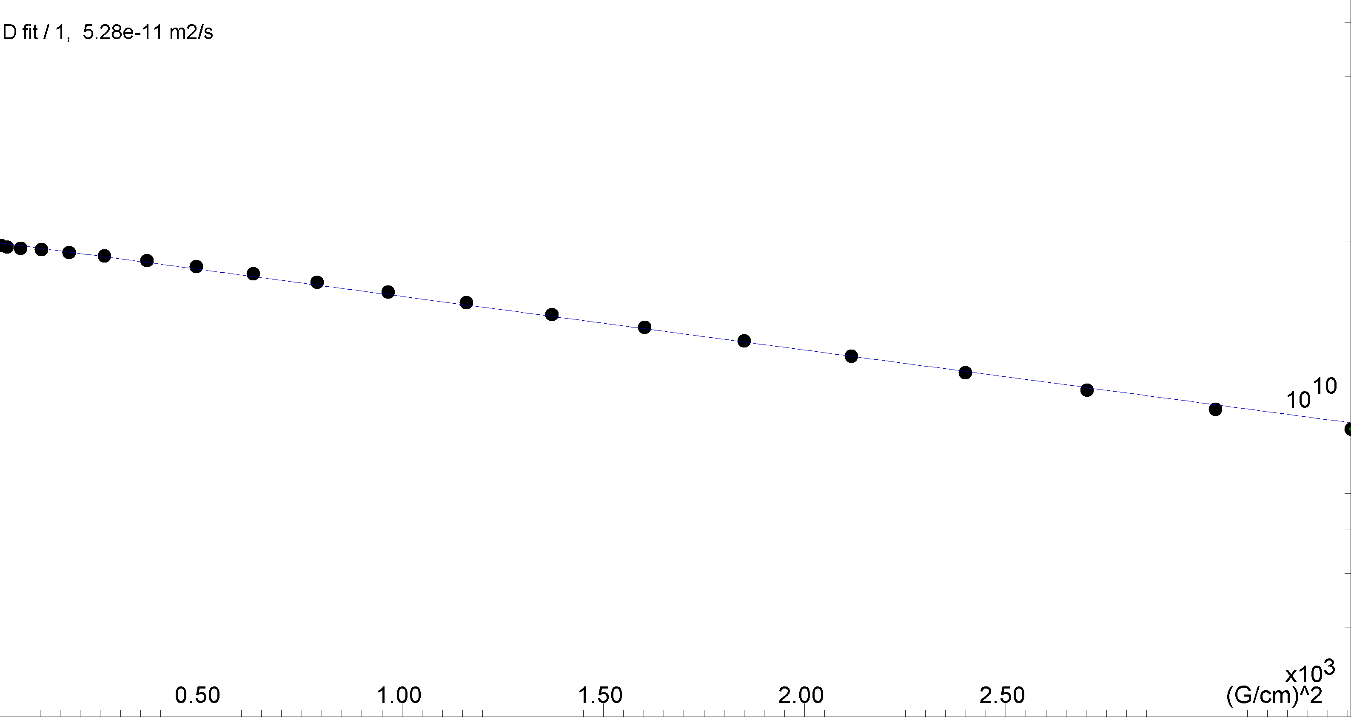


1. **DPC Methylene region (3.53-3.51 ppm)**

results of peak 2

ppm 3.523

fitted function f (x) = Io*exp (-D*x^2 *gamma^2 * littleDelta^2 (bigDelta-littleDelta/3)* 10^4

calculated Io 1.317e+09 error 1.950e+05

calculated D 7.878e-11 error 3.621e-14 m2/s

used gamma: 26752 rad/(s*Gauss)

used little delta: 0.0020000 s

used big delta: 0.14990 s

used gradient strength: variable

x variables all variables used even if identical

fit points all non-zero integrals/volumes used

numerical values

X variable [G/cm] original Y fitted Y residual

1.1830 1.3454e+09 1.3159e+09 2.9447e+07

4.1700 1.3268e+09 1.3088e+09 1.7931e+07

7.1580 1.3007e+09 1.2940e+09 6.7182e+06

10.145 1.2731e+09 1.2717e+09 1.4069e+06

13.133 1.2378e+09 1.2423e+09 -4.4711e+06

16.121 1.1976e+09 1.2063e+09 -8.6946e+06

19.108 1.1534e+09 1.1643e+09 -1.0880e+07

22.096 1.1069e+09 1.1170e+09 -1.0165e+07

25.084 1.0535e+09 1.0653e+09 -1.1741e+07

28.071 9.9674e+08 1.0099e+09 -1.3118e+07

31.059 9.3930e+08 9.5156e+08 -1.2257e+07

34.046 8.7891e+08 8.9127e+08 -1.2364e+07

37.034 8.1976e+08 8.2979e+08 -1.0032e+07

40.022 7.6512e+08 7.6792e+08 -2.7972e+06

43.009 7.1010e+08 7.0642e+08 3.6713e+06

45.997 6.5273e+08 6.4594e+08 6.7869e+06

48.984 5.9510e+08 5.8712e+08 7.9844e+06

51.972 5.3925e+08 5.3044e+08 8.8130e+06

54.960 4.8740e+08 4.7636e+08 1.1037e+07

57.947 4.3715e+08 4.2525e+08 1.1906e+07

Goodness of fit:

SSE (final chi) 40169.7990

R-square 0.9984

R-square adjusted 0.9983

RMSE 47.2404

2-sided ShapiroWilk test 0.1062

Interpretation Normal distribution of residuals likely


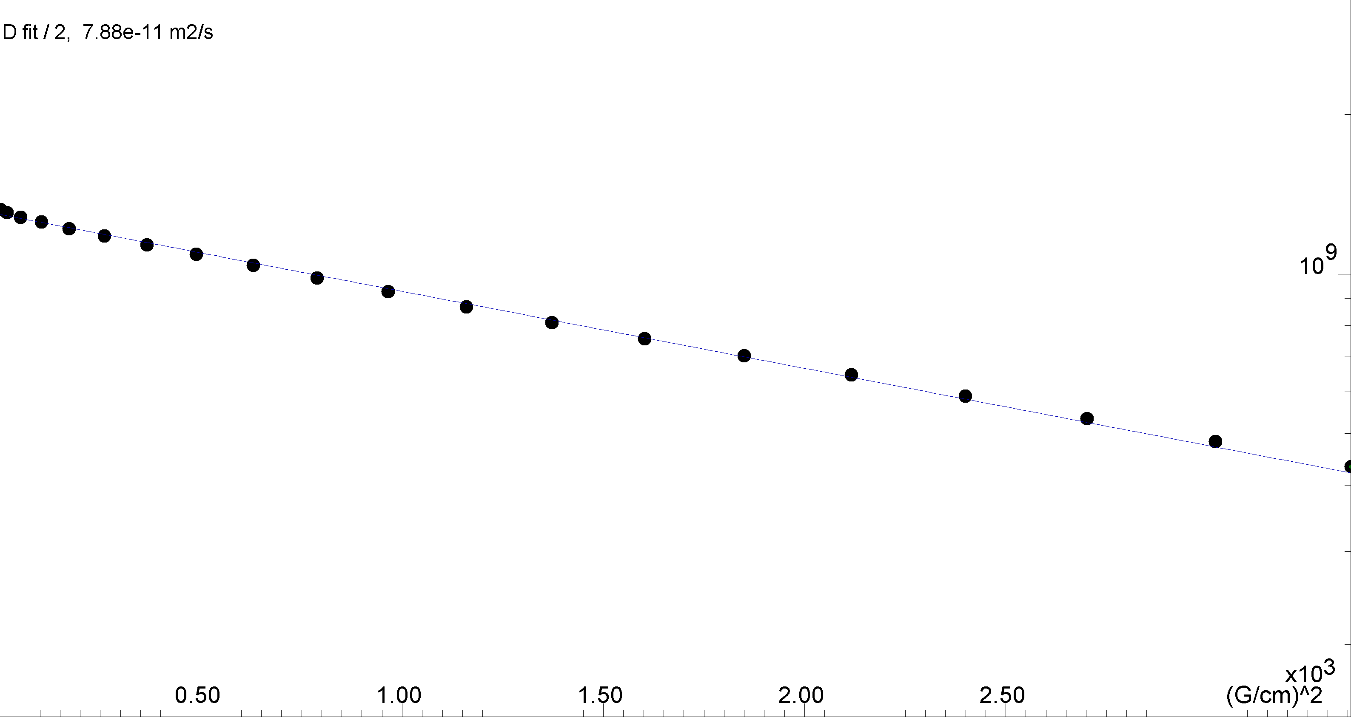


1. **DPC Methylene region (1.09-1.17 ppm)**

results of peak 3

ppm 1.129

fitted function f (x) = Io * exp (-D * x^2 * gamma^2 * littleDelta^2 (bigDelta-littleDelta/3)* 10^4

best fit with 2 function terms

calculated Io 2.62e+10 error 1.611e+07

calculated D 8.24e-11 error 7.956e-14

calculated Io 1.25e+09 error 4.921e+06

calculated D 1.34e-09 error 1.572e-23

used gamma: 26752 rad/(s*Gauss)

used little delta: 0.0020000 s

used big delta: 0.14990 s

used gradient strength: variable

x variables all variables used even if identical

fit points all non-zero integrals/volumes used

numerical values

X variable [G/cm] original Y fitted Y residual

1.1830 2.7562e+10 2.7433e+10 1.2928e+08

4.1700 2.7129e+10 2.7178e+10 -4.8421e+07

7.1580 2.6563e+10 2.6671e+10 -1.0821e+08

10.145 2.5939e+10 2.5969e+10 -3.0492e+07

13.133 2.5138e+10 2.5131e+10 7.8253e+06

16.121 2.4231e+10 2.4200e+10 3.0413e+07

19.108 2.3253e+10 2.3202e+10 5.0850e+07

22.096 2.2198e+10 2.2146e+10 5.1934e+07

25.084 2.1063e+10 2.1035e+10 2.8465e+07

28.071 1.9886e+10 1.9872e+10 1.3933e+07

31.059 1.8636e+10 1.8665e+10 -2.8801e+07

34.046 1.7381e+10 1.7426e+10 -4.5234e+07

37.034 1.6114e+10 1.6169e+10 -5.5657e+07

40.022 1.4864e+10 1.4910e+10 -4.5230e+07

43.009 1.3636e+10 1.3662e+10 -2.5873e+07

45.997 1.2424e+10 1.2441e+10 -1.6464e+07

48.984 1.1259e+10 1.1258e+10 1.4245e+06

51.972 1.0145e+10 1.0123e+10 2.1966e+07

54.960 9.0865e+09 9.0454e+09 4.1086e+07

57.947 8.0935e+09 8.0323e+09 6.1182e+07

Goodness of fit:

SSE (final chi) 218043.1181

R-square 0.9999

R-square adjusted 0.9999

RMSE 116.7377

2-sided ShapiroWilk test 0.9321

Interpretation Normal distribution of residuals likely


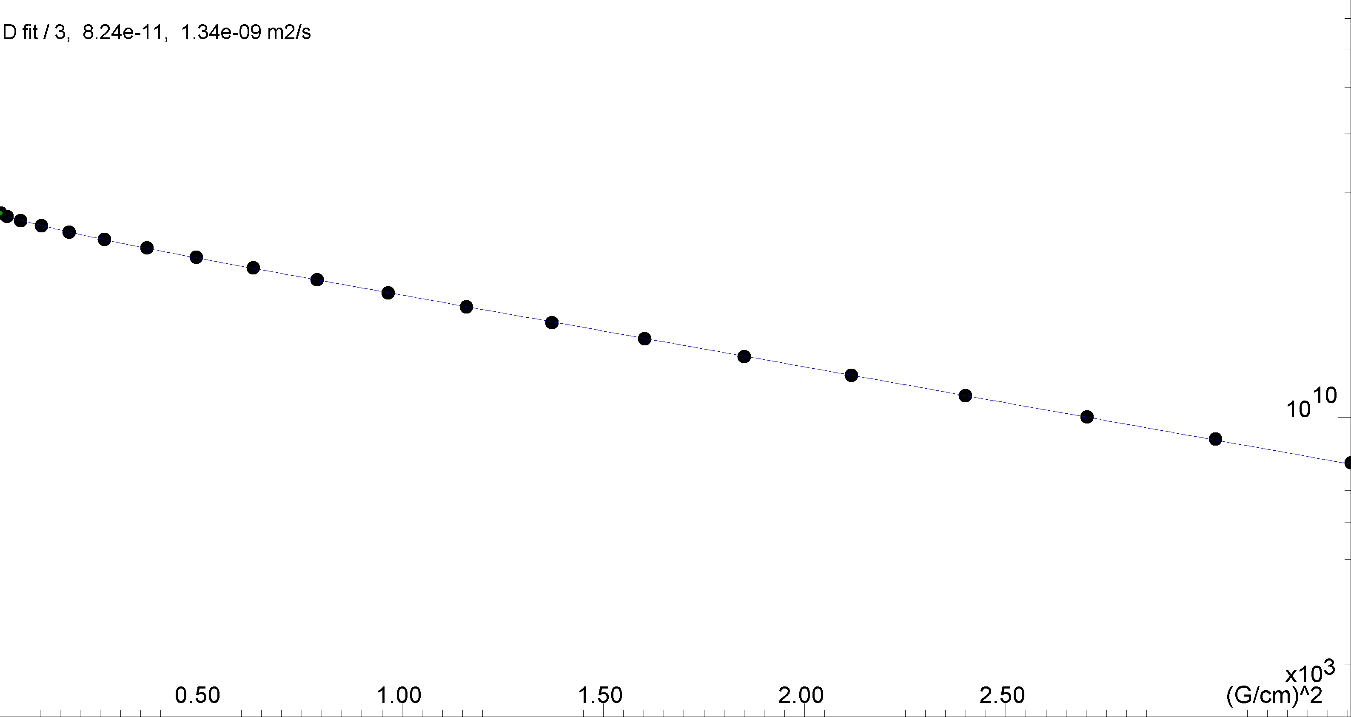


**Figure S7**. Dynamics Center Properties of D fit of DT-P2 sample

DT-P2 sample: 0.5 mM DT-P2 in 10 mM phosphate buffer (pH = 6.0, in 100 % D_2_O: 0.5 mM DSS and 1.0 mM TCEP) with 50 mM DPC-d_38_.

1. **DT-P2 Aromatic Region (6.65-7.48 ppm)**

results of peak 1

ppm 7.066

fitted function f (x) = Io * exp (-D * x^2 * gamma^2 * littleDelta^2 (bigDelta-littleDelta/3)* 10^4

calculated Io 3.890e+10 error 1.255e+06

calculated D 6.940e-11 error 7.633e-15 m2/s

used gamma: 26752 rad/(s*Gauss)

used little delta: 0.0020000 s

used big delta: 0.14990 s

used gradient strength: variable

x variables all variables used even if identical

fit points all non-zero integrals/volumes used

numerical values

X variable [G/cm] original Y fitted Y residual

1.1830 3.9360e+10 3.8881e+10 4.7910e+08

4.1700 3.8903e+10 3.8697e+10 2.0659e+08

7.1580 3.8417e+10 3.8310e+10 1.0621e+08

10.145 3.7749e+10 3.7728e+10 2.1671e+07

13.133 3.6907e+10 3.6958e+10 -5.0902e+07

16.121 3.5908e+10 3.6012e+10 -1.0458e+08

19.108 3.4756e+10 3.4906e+10 -1.5014e+08

22.096 3.3488e+10 3.3655e+10 -1.6739e+08

25.084 3.2081e+10 3.2277e+10 -1.9575e+08

28.071 3.0612e+10 3.0793e+10 -1.8143e+08

31.059 2.9085e+10 2.9222e+10 -1.3641e+08

34.046 2.7454e+10 2.7584e+10 -1.3043e+08

37.034 2.5797e+10 2.5901e+10 -1.0374e+08

40.022 2.4138e+10 2.4192e+10 -5.3549e+07

43.009 2.2421e+10 2.2477e+10 -5.5923e+07

45.997 2.0750e+10 2.0773e+10 -2.2846e+07

48.984 1.9147e+10 1.9097e+10 4.9854e+07

51.972 1.7614e+10 1.7463e+10 1.5147e+08

54.960 1.6086e+10 1.5885e+10 2.0126e+08

57.947 1.4617e+10 1.4373e+10 2.4427e+08

Goodness of fit:

SSE (final chi) 193688.5947

R-square 0.9995

R-square adjusted 0.9995

RMSE 103.7327

2-sided ShapiroWilk test 0.07150

Interpretation Normal distribution of residuals likely

**
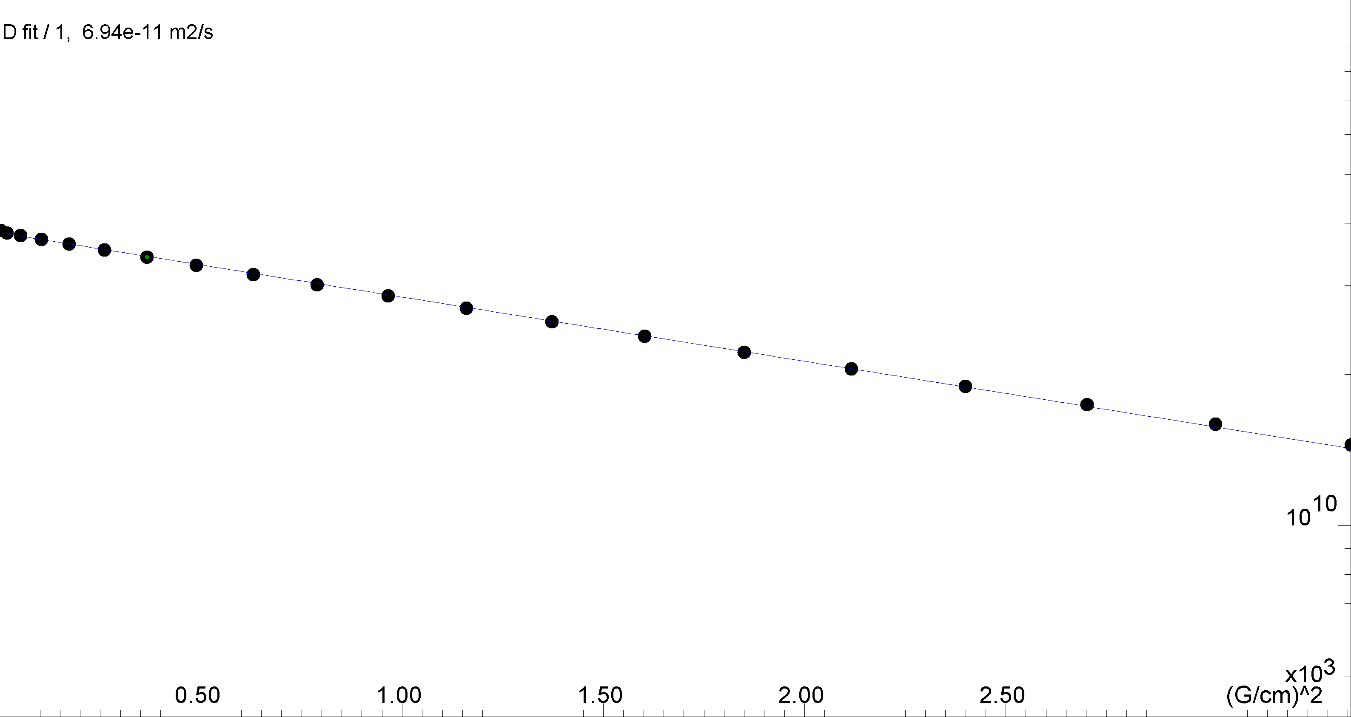
**

1. **DPC Methylene region (3.49-3.53 ppm)**

results of peak 4

ppm 3.513

fitted function f (x) = Io * exp (-D * x^2 * gamma^2 * littleDelta^2 (bigDelta-littleDelta/3)* 10^4

calculated Io 1.792e+09 error 3.016e+05

calculated D 8.928e-11 error 4.389e-14 m2/s

used gamma: 26752 rad/(s*Gauss)

used little delta: 0.0020000 s

used big delta: 0.14990 s

used gradient strength: variable

x variables all variables used even if identical

fit points all non-zero integrals/volumes used

numerical values

X variable [G/cm] original Y fitted Y residual

1.1830 1.8154e+09 1.7909e+09 2.4452e+07

4.1700 1.7958e+09 1.7800e+09 1.5752e+07

7.1580 1.7624e+09 1.7572e+09 5.2186e+06

10.145 1.7225e+09 1.7229e+09 -3.9245e+05

13.133 1.6698e+09 1.6778e+09 -7.9983e+06

16.121 1.6120e+09 1.6228e+09 -1.0816e+07

19.108 1.5455e+09 1.5589e+09 -1.3443e+07

22.096 1.4756e+09 1.4874e+09 -1.1844e+07

25.084 1.4000e+09 1.4096e+09 -9.5709e+06

28.071 1.3195e+09 1.3267e+09 -7.2971e+06

31.059 1.2365e+09 1.2403e+09 -3.7996e+06

34.046 1.1532e+09 1.1516e+09 1.5394e+06

37.034 1.0676e+09 1.0620e+09 5.5890e+06

40.022 9.8154e+08 9.7274e+08 8.7969e+06

43.009 8.8917e+08 8.8495e+08 4.2257e+06

45.997 7.9115e+08 7.9959e+08 -8.4359e+06

48.984 7.0640e+08 7.1758e+08 -1.1183e+07

51.972 6.4053e+08 6.3960e+08 9.3161e+05

54.960 5.7556e+08 5.6622e+08 9.3444e+06

57.947 5.1534e+08 4.9788e+08 1.7463e+07

Goodness of fit:

SSE (final chi) 14989.4550

R-square 0.9994

R-square adjusted 0.9993

RMSE 28.8574

2-sided ShapiroWilk test 0.3668

Interpretation Normal distribution of residuals likely

**
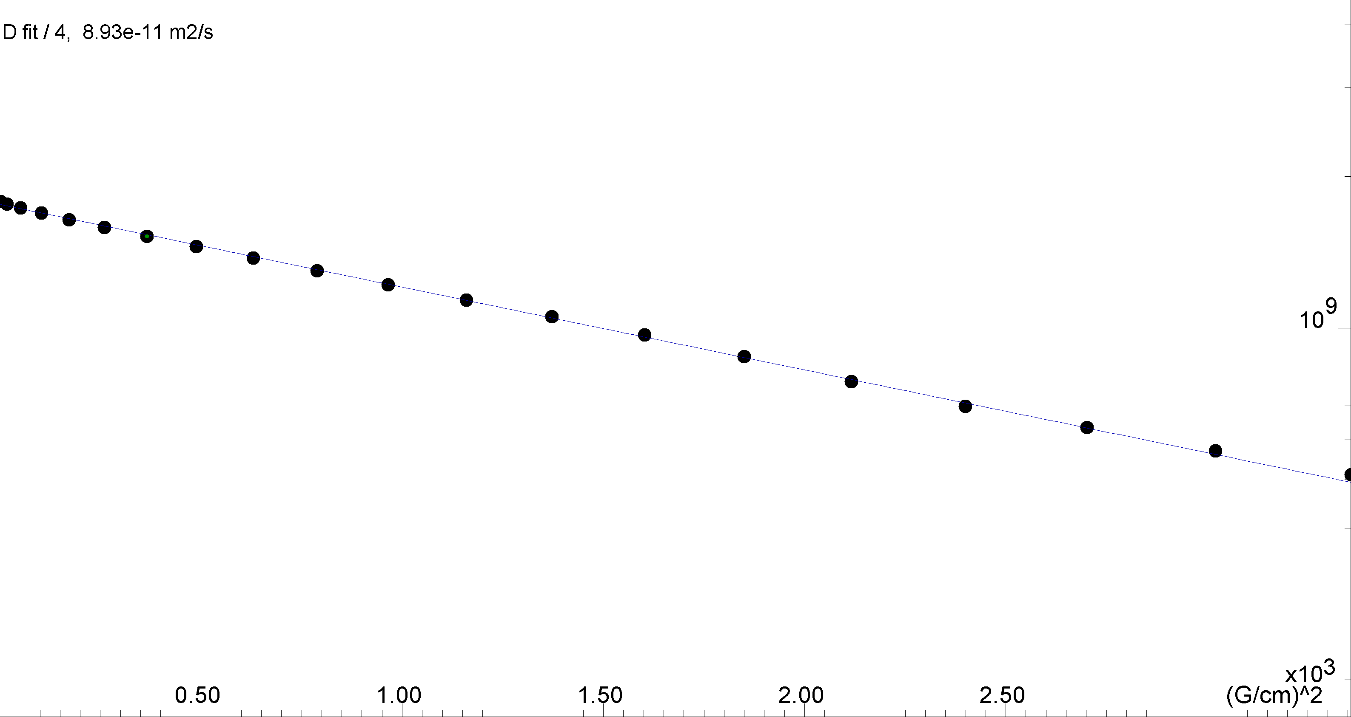
**

1. **DPC Methylene region (1.08-1.16 ppm)**

results of peak 2

ppm 1.119

fitted function f (x) = Io * exp (-D * x^2 * gamma^2 * littleDelta^2 (bigDelta-littleDelta/3)* 10^4

best fit with 2 function terms

calculated Io 2.30e+10 error 1.837e+06

calculated D 8.90e-11 error 1.027e-14

calculated Io 4.80e+09 error 1.779e+06

calculated D 9.39e-10 error 6.285e-13

used gamma: 26752 rad/(s*Gauss)

used little delta: 0.0020000 s

used big delta: 0.14990 s

used gradient strength: variable

x variables all variables used even if identical

fit points all non-zero integrals/volumes used

numerical values

X variable [G/cm] original Y fitted Y residual

1.1830 2.8173e+10 2.7752e+10 4.2103e+08

4.1700 2.7222e+10 2.7316e+10 -9.3642e+07

7.1580 2.6177e+10 2.6456e+10 -2.7855e+08

10.145 2.5043e+10 2.5285e+10 -2.4191e+08

13.133 2.3828e+10 2.3935e+10 -1.0652e+08

16.121 2.2561e+10 2.2520e+10 4.1345e+07

19.108 2.1264e+10 2.1120e+10 1.4407e+08

22.096 1.9960e+10 1.9772e+10 1.8794e+08

25.084 1.8635e+10 1.8483e+10 1.5190e+08

28.071 1.7325e+10 1.7241e+10 8.4601e+07

31.059 1.6039e+10 1.6030e+10 9.3143e+06

34.046 1.4763e+10 1.4840e+10 -7.7334e+07

37.034 1.3525e+10 1.3665e+10 -1.4018e+08

40.022 1.2345e+10 1.2509e+10 -1.6481e+08

43.009 1.1181e+10 1.1379e+10 -1.9765e+08

45.997 1.0096e+10 1.0282e+10 -1.8636e+08

48.984 9.1669e+09 9.2300e+09 -6.3085e+07

51.972 8.3072e+09 8.2292e+09 7.7965e+07

54.960 7.4715e+09 7.2874e+09 1.8417e+08

57.947 6.6806e+09 6.4099e+09 2.7069e+08

Goodness of fit:

SSE (final chi) 2368760.5235

R-square 0.9993

R-square adjusted 0.9992

RMSE 384.7695

2-sided ShapiroWilk test 0.7486

Interpretation Normal distribution of residuals likely
